# Nephron-Specific *Lin28A* Overexpression Triggers Severe Inflammatory Response and Kidney Damage

**DOI:** 10.7150/ijbs.97434

**Published:** 2024-07-22

**Authors:** Anna Futorian, Leah Armon, Hiba Waldman Ben-Asher, Irit Shoval, Inbal Hazut, Ariel Munitz, Achia Urbach

**Affiliations:** 1 The Mina and Everard Goodman Faculty of Life Sciences, Bar-Ilan University, Ramat Gan, Israel.; 2 Department of Clinical Microbiology & Immunology, Faculty of Medical and Health Sciences, Tel Aviv University, Tel Aviv, Israel.

## Abstract

The RNA-binding proteins LIN28A and LIN28B contribute to a variety of developmental biological processes. Dysregulation of *Lin28A* and *Lin28B* expression is associated with numerous types of tumors. This study demonstrates that *Lin28A* overexpression in the mouse nephrons leads to severe inflammation and kidney damage rather than to tumorigenesis. Notably, *Lin28A* overexpression causes inflammation only when expressed in nephrons, but not in the stromal cells of the kidneys, highlighting its cell context-dependent nature. The nephron-specific *Lin28A*-induced inflammatory response differs from previously described *Lin28B*-mediated inflammatory feedback loops as it is IL-6 independent. Instead, it is associated with the rapid upregulation of cytokines like *Cxcl1* and *Ccl2*. These findings suggest that the pathophysiological effects of *Lin28A* overexpression extend beyond cell transformation. Our transgenic mouse model offers a valuable tool for advancing our understanding of the pathophysiology of acute kidney injury, where inflammation is a key factor.

## Introduction

*Lin28*, initially identified as a heterochronic gene in Caenorhabditis elegans, is an RNA-binding protein^1^,^2^ that regulates gene expression through two distinct mechanisms: one blocks maturation of the *Let-7* microRNA (miRNA) family^3^-^6^, preventing gene translation suppression by these miRNA^6^-^8^; the other, mediated via a *Let-7*-independent pathway, directly binds to mRNAs and modifies gene expression^8^-^10^.

In mammals, there are two paralogs, *Lin28A* and *Lin28B,* which are expressed mainly in pluripotent and multipotent stem cells. *Lin28A* and *Lin28B (*henceforth,* Lin28A/B)* play a role in various biological processes, including development, growth, metabolism, tissue regeneration, and others^7^,^8^,^11^-^18^, via the regulation of diverse molecular pathways (for review, see^7^,^8^,^12^). For example, *Lin28A/B* regulate the activity of the mTOR pathway to promote the proliferation of neuronal progenitor cells^19^, and to regulate glucose metabolism^13^. Normally, *Lin28A/B* undergo downregulation upon cell differentiation and their ectopic/overexpression is associated with numerous types of pediatric and adult tumors^7^,^8^,^12^,^20^-^24^.

Despite the high similarity between these paralogous genes and their common function in blocking *Let-7* maturation, these proteins differ in their cellular localization, mechanisms of *Let-7* inhibition, regulatory signals governing their expression, and spatial distribution (for reviews, see^8^,^12^,^25^). A significant piece of evidence supporting the distinct roles and functions of these two paralogous proteins is the pronounced difference in phenotypes observed among *Lin28A* knockout (KO) mice, *Lin28B* KO mice, and *Lin28A/B* double KO mice^26^. Additionally, some roles have been demonstrated only for one of the two paralogs. For example, it has been shown that *Lin28A* interacts with signaling factors of the FGF/WNT, SHH, and BMP pathways to control the elongation of the caudal body axis^15^, and that *Lin28B* regulates mTOR pathway activity to enable the generation of cochlear hair cells^27^. *Lin28B* overexpression (OE) was also shown to play a role in a positive feedback loop connecting inflammatory response and hematopoietic cancer formation via the NF-κB-LIN28B-IL-6-STAT3 pathway^28^.

Both* Lin28A* and* Lin28B* are expressed during mouse embryonic kidney development in the nephron progenitor cap-mesenchyme (CM) cells until E13.5 and E16.5, respectively^16^,^17^. We and others found that *Lin28A/B* regulate the balance between CM proliferation and differentiation and are required for normal nephrogenesis^16^,^17^. Specifically, we showed that *Lin28A/B* OE during embryonic nephrogenesis in the entire kidney led to abnormal CM proliferation and development of a kidney tumor highly resembling Wilms tumor^16^. Interestingly, however, specific *Lin28A/B* OE in the CM cells did not result in abnormal CM proliferation but led to the development of severe postnatal kidney damage^16^. This effect was not due to abnormal embryonic kidney development, as *Lin28A/B* OE in adult nephrons produced a similar phenotype^16^.

In the current study, we studied this pronounced effect of *Lin28A/B* OE on the adult kidney, focusing on *Lin28A*. Remarkably, we found that the severe kidney damage resulted from an inflammatory response induced by the overexpression of *Lin28A*. We further found that the *Lin28A*-induced inflammatory response is associated with abnormal activation of the AKT pathway. These findings extend our understanding of *Lin28A* OE impact, showing that under a specific cellular context, *Lin28* OE leads to an inflammatory response rather than tumorigenesis.

## Materials and Methods

### Mice

All procedures were approved by the University of Bar-Ilan Institutional Animal Care and Use Committee. The generation and maintenance of TRE-Lin28A mice were previously described^11^,^13^,^14^,^16^. These mice were crossed with Rosa26-Lox-stop-Lox mice, as described in 11,13,14,16**,** to obtain homozygous TRE-*Lin28A*;Lox-stop-Lox-TetOn-rtTA. Homozygous TRE-*Lin28A*;Lox-stop-Lox-TetOn-rtTA were crossed with Six2Cre mice (Jackson Laboratories Stock #009606) or FoxD1Cre mice (Jackson Laboratories Stock #012463) to achieve nephron and stromal (respectively) cell-specific *Lin28A* OE upon Dox treatment. Doxycycline - Doxycycline (Sigma-Aldrich; 1g/L) was administered to the drinking water for different time durations, as described in the Results section. Rapamycin - Rapamycin (LC Laboratories; 4 mg/kg mouse weight) was injected intraperitoneally thrice weekly for three weeks (starting at P28). Dexamethasone - Dexamethasone (Sigma-Aldrich; 25μg) was injected intraperitoneally three times a week for a three-week period. In addition, 20 mg/L of Dexamethasone was supplemented in the drinking water.

**Histology and Immunofluorescence staining:** Samples were fixed with 4% paraformaldehyde diluted in PBS, dehydrated in 75% EtOH, and embedded in paraffin. Sections were cut 7-μm thick and dried overnight at 37°C. The slides were dewaxed with xylene and rehydrated through a series of washes with decreasing percentages of ethanol. Antigen retrieval was performed in 10 mM sodium citrate buffer (pH 6.0) by placing it in a pressure cooker for 15 min at a high temperature. Sections were blocked in 10% serum and incubated overnight at 4°C with the primary antibodies. Tissue sections were washed with PBS and incubated for 1 h at room temperature with secondary antibodies. Sections were treated with DAPI before mounting with mounting solution (Fluoromount-G, Thermo Fisher Scientific). For the list of antibodies, see **Table S1**.

For hematoxylin and eosin (H&E) staining, sections were paraffin-embedded. Next, the sections were stained with Harris hematoxylin solution (Kaltek) for 1 min and with eosin (Millipore) for 15 s. Mounting was conducted with DPX Mountant (Millipore).

**RNA extraction and qRT-PCR analysis:** Total RNA was extracted using TRI Reagent (RiboEx, GeneALL). cDNA was synthesized using iScript cDNA Synthesis kit (Bio-Rad). qRT-PCR analysis was performed using PerfecTa SYBR Green FastMix (Quantabio). β-actin was used for normalization. Relative expression was analyzed using the 2^-ΔΔCt^ method. The data are represented as relative expression (fold change); however, the statistical analysis was performed on the ΔΔCt values due to their linearity. For primer list, see **Table S1**.

**Western Blot:** Total protein was extracted from harvested kidneys. Kidneys were collected and snap-frozen in liquid nitrogen. Next, 10-20mg tissue pieces were homogenized using Bullet Blender homogenizer (Next Advance) in ice-cold lysis buffer (10 mM TrisHCl pH 7.4, 150 mM NaCl, 1 mM EDTA, 1 mM EGTA, 0.5% TritonX100, 7 M urea, protease inhibitor cocktail (Millipore), and phosphatase inhibitor cocktail (Cell Signaling)). The extracted proteins were separated by 10% acrylamide gel, followed by transfer to a nitrocellulose membrane. The membranes were blocked with 2% bovine serum albumin in TBST (TBS with 0.05% Tween20). Then, the membranes were incubated with the appropriate antibodies and developed with EZ-ECL (Biological Industries). For the list of antibodies, see **Table S1**.

**Analysis of WBC and BUN levels in the blood:** WBC count was performed using the Exigo™ H400 veterinary hematology analyzer (Boule), according to the manufacturer's instructions. BUN levels were measured using the Vetscan 2 (Zoetis), according to the manufacturer's instructions.

**Flow Cytometry:** The kidneys were obtained from lin28A OE and littermate control mice and mashed through a 100μm strainer (431752, Corning). Cell suspensions were collected and incubated for 10 minutes with RBC lysis buffer. Staining was performed on ice after incubation with anti-CD16/CD32 (14-0161-82, eBioscience) to reduce non-specific binding to Fc receptors. Flow cytometric analysis was performed using Gallios (Beckman-Coulter). To obtain absolute cell counts, Flow-Count Fluorosphers (#7547053, Beckman-Coulter) were used according to the manufacturer's instructions. Data were analyzed using Kaluza analysis software (Beckman-Coulter). For the list of the fluorochrome-labeled anti-mouse antibodies used, see **Table S1.**

**ELISA:** Protein levels of TNF-α, IL-6 and CCL2 were determined using commercially available kits (TNF-α: DY410, R&D; CCL2: DY479, R&D; and IL-6: 900-T50, Peprotech), according to the manufacturer's instructions.

**RNA seq:** RNA quality control - For QC of purified RNA, absorbance ratios A260:A280 and A260:A230 were assessed with NanoDrop 2000. The integrity of RNA was evaluated based on RIN acquired via capillary gel electrophoresis performed using Agilent 4200 TapeStation in combination with Agilent RNA ScreenTape System (Agilent Technologies). All RNA samples went through Dnase Treatment Kit (Qiagen) before proceeding to the next step. PolyA selection and library preparation - For library preparation, NEBNext RNA ultra II RNA library preparation kit (NEB) was used. All RNA samples underwent PolyA selection following the manufacturer's protocols. Samples were multiplexed using suitable molecular barcodes, and resulting cDNA pools were processed according to the NextSeq System Denature and Dilute Libraries guide. (Illumina). Quantification and quality control of the libraries were done using a Qubit fluorimeter and Agilent 4200 TapeStation. Next-generation sequencing - Single-read sequencing of the libraries with a read length of 75 was performed with NextSeq 500 Sequencing System using NextSeq 500/550 High Output v2 kit (75 cycles) (20024906 Illumina). PhiX Control v3 (Illumina) was added at 1% to all pools as an internal control before the sequencing. The RNA seq data has been deposited in Gene Expression Omnibus (GEO), series accession number GSE254805.

**Bioinformatics analysis:** Sequenced reads were mapped to the mouse reference genome sequences (mm10) using STAR. The aligned reads were quantitated by Htseq. The normalization and differentially expressed genes test were implemented by DESeq2. For Pathways and GO terms enriched analysis, the DEG list was analyzed by GeneAnalytics (https://geneanalytics.genecards.org/).

**Statistical analysis:** All data were generated from at least three independent experiments. P values of less than 0.05 were considered statistically significant. All analyses were performed using the GraphPad Prism software. For additional information regarding the precise analyses used, see figure legends.

## Results

### Nephron-specific *Lin28A* OE leads to rapid damage in diverse types of kidney cells

**Figure 1:**
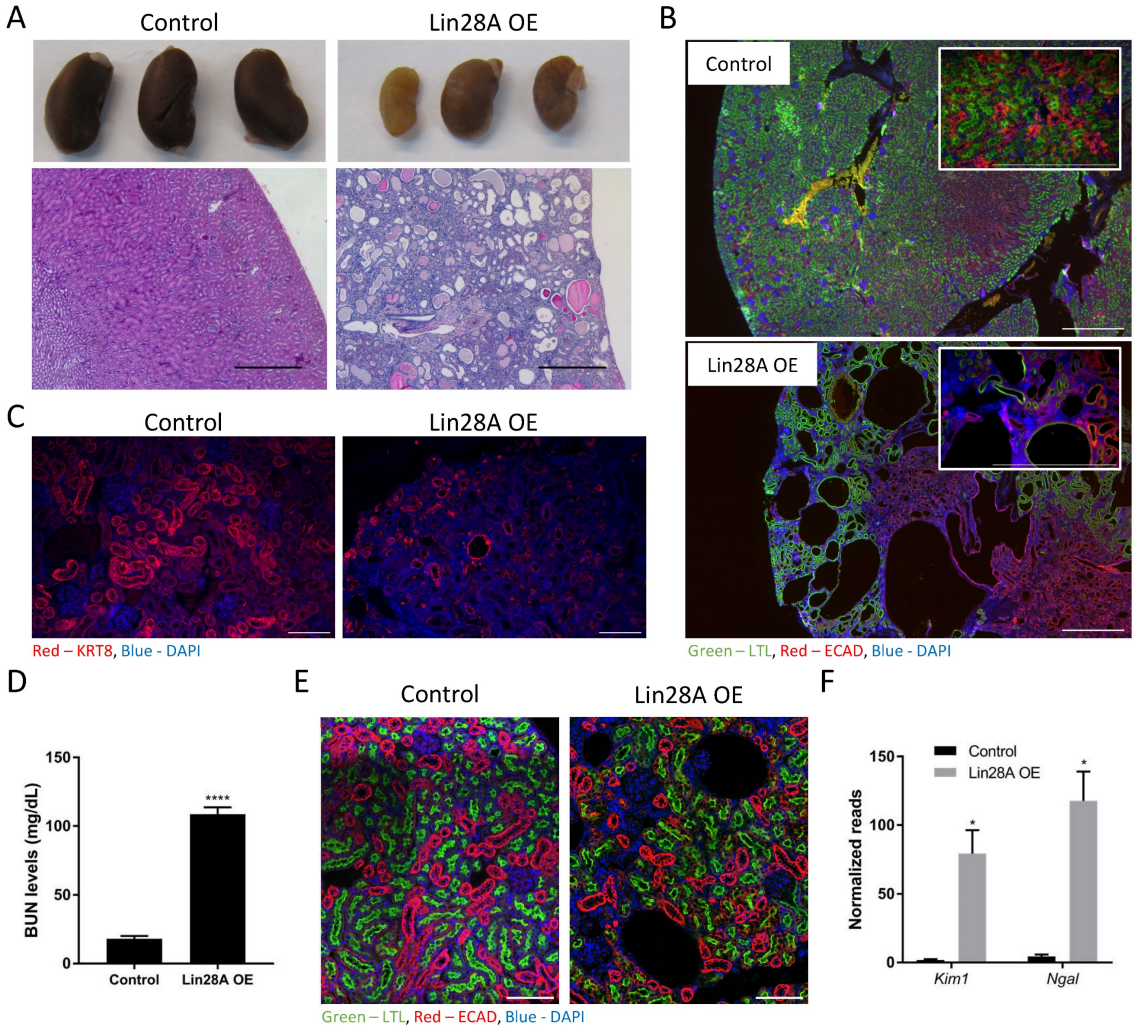
** Nephron-specific *Lin28A* OE leads to severe kidney damage. A.** Gross morphology (upper panel) and H&E staining (lower panel) of control and *Lin28A* OE kidneys following 11 weeks of* Lin28A* OE induction. Scale bar - 500µM. **B.** Control and *Lin28A* OE kidneys stained for PT (LTL) and DT/CD (ECAD) following 5 weeks of *Lin28A* OE induction. Scale bar - 500µM. **C.** Control and *Lin28A* OE kidneys stained for CD (KRT8) following 5 weeks of *Lin28A* OE induction. Scale bar - 100µM. **D.** BUN levels in control and Lin28 OE kidneys upon 3 weeks of Dox treatment. **E.** Control and Lin28 OE kidneys stained for PT (LTL) and DT/CD (ECAD) upon 1 week of Dox treatment. Scale bar - 100µM. **F.** RNA seq normalized read counts of *Kim1* and *Ngal* in *Lin28A* OE kidneys compared to control kidneys upon 1 week of Dox treatment. Statistical analysis using Student's t-test (D) or Multiple t-tests with Holm-Sidak post-test. (F). N=3. * P<0.05. **** P<0.0001.

We have shown previously that *Lin28A/B* OE in the nephrons causes severe kidney damage^16^. To further explore this effect, we used the same TRE-*Lin28A*;Lox-stop-Lox-TetOn-rtTA transgenic mice described previously^11^,^14^,^16^. Crossing this strain with *Six2***-**Cre mice removes the stop cassette specifically in the CM cells and their derivates, i.e., the adult nephrons. This enables a specific *Lin28A* OE upon doxycycline (Dox) treatment, which leads to severe kidney damage (**Figure 1A**). To study if the kidney damage is specific to the nephron or if *Lin28A* OE in the nephrons affects other cell types in the kidney, we performed immunofluorescent staining with markers for proximal tubules (PT), distal tubules (DT), and collecting duct (CD). Interestingly, we found that nephron-specific *Lin28A* OE damaged not only the PT and the DT but also the CD, which is derived from the ureteric bud lineage and not from the *Six2* positive nephron progenitor cells^29^ (**Figure 1B,C and Figure S1A**). This observation suggests that nephron-specific *Lin28A* OE has a broad effect on the kidneys. The increased blood urea nitrogen (BUN) levels (**Figure 1D**) further confirm the severe damage caused by *Lin28A* OE. Notably, this effect of *Lin28A* OE emerges within just one week, as evidenced by H&E (**Figure S1B**) and immunostaining (**Figure 1E**). To further characterize the kidney damage and to explore its cause, we performed bulk RNA sequencing on kidneys following one, two, and five weeks of Dox treatment. The increased levels of kidney injury markers such as *Kim1* and *Ngal* 30,31 already shown after one week of Dox treatment (**Figure 1F**) further demonstrate the pronounced and rapid kidney damage.

### *Lin28A* OE in the nephrons triggers an inflammatory response

**Figure 2:**
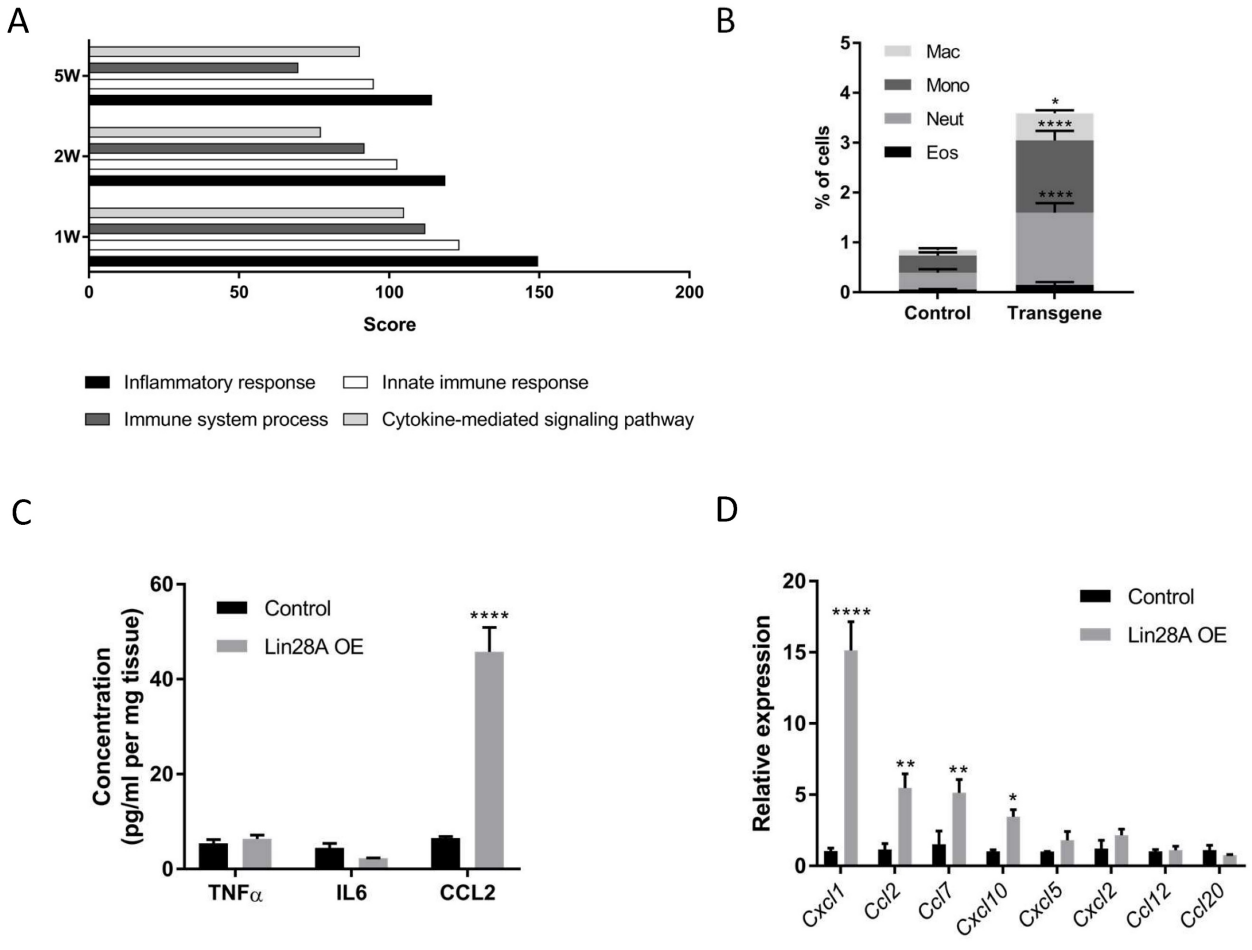
** Nephron-specific *Lin28A* OE triggers an inflammatory response. A.** Top GO terms categories of upregulated genes upon 1, 2, and 5 weeks of *Lin28A* OE. **B.** FACS analysis of immune cells in the transgenic and control kidneys. Mac - macrophages, Mono - monocytes, Neut - neutrophils, Eos - eosinophils. **C.** CCL2, IL-6, and TNFα protein levels as detected by ELISA upon 3 weeks of Dox treatment. **D.** qRT-PCR analysis for cytokines expression levels (normalized to βActin) upon 48 hours of Dox treatment. Statistical analysis using 2-way ANOVA with Sidak post-test (B, D) or multiple t-tests with Holm-Sidak post-test (C). N=3 for panels A,B,D. N=6 for panel C. * P<0.05, **P<0.01, ***P<0.001, **** P<0.0001.

Principal Component Analysis (PCA) of the RNA sequencing results indicates that while all the control kidneys form a cohesive cluster, each group of *Lin28A* OE kidneys forms a distinct cluster (**Figure S2A**). Notably, however, analysis of the differentially expressed genes (DEGs, defined based on the following criteria: Log2fold > 1 or < -1; Baseman > 10; and adjusted P-value <0.05) revealed that the top GO terms associated with the *Lin28A* OE upregulated DEGs were all related to inflammatory response in all the *Lin28A* OE groups (**Figure 2A**). This unexpected observation suggests that *Lin28A* OE in the nephrons induces an inflammatory response. To validate this finding, we performed FACS analysis for different types of immune cells (**Figure 2B**). We further conducted ELISA, which revealed upregulation of CCL2 but not of IL-6 or TNFα (**Figure 2C**). Consistent with the ELISA results, the RNA seq also showed significant upregulation of *Ccl2* (**Figure S2B**). However, contrary to the ELISA results, there was upregulation of *Tnfα* at the RNA level (**Figure S2B**).

We further explored if the inflammatory response was the direct effect of *Lin28A* OE or a secondary effect triggered by another kidney damage. To this end, we first generated a list of the most upregulated cytokines upon one week of Dox induction **Table S2**. Next, we analyzed the expression of these cytokines 48h following Dox induction (which is 24h subsequent to *Lin28A* upregulation (**Figure S2C**)). The rapid upregulation of some of these cytokines (**Figure 2D**) suggests that the inflammatory response is a direct effect of *Lin28A* OE.

As expected, DEG analysis between two weeks and one week of Dox induction showed that the trend of upregulation in genes and pathways related to the immune system was maintained between the first and the second week (**Figure S3**). Similarly, we found a trend of downregulation in genes related to metabolic pathways, indicating the worsening of kidney damage over time. Interestingly, we found only a handful of genes that changed their gene expression dynamics (from upregulation after one week to downregulation after two weeks and vice versa). It appears that this change has no functional effect, as these genes are not part of any specific pathway or GO term (**Figure S3**), and no one of them by itself is expected to play a role in the kidney phenotype (for the lists of all groups of DEGs representing gene expression dynamic, see **Table S3**).

**Figure 3:**
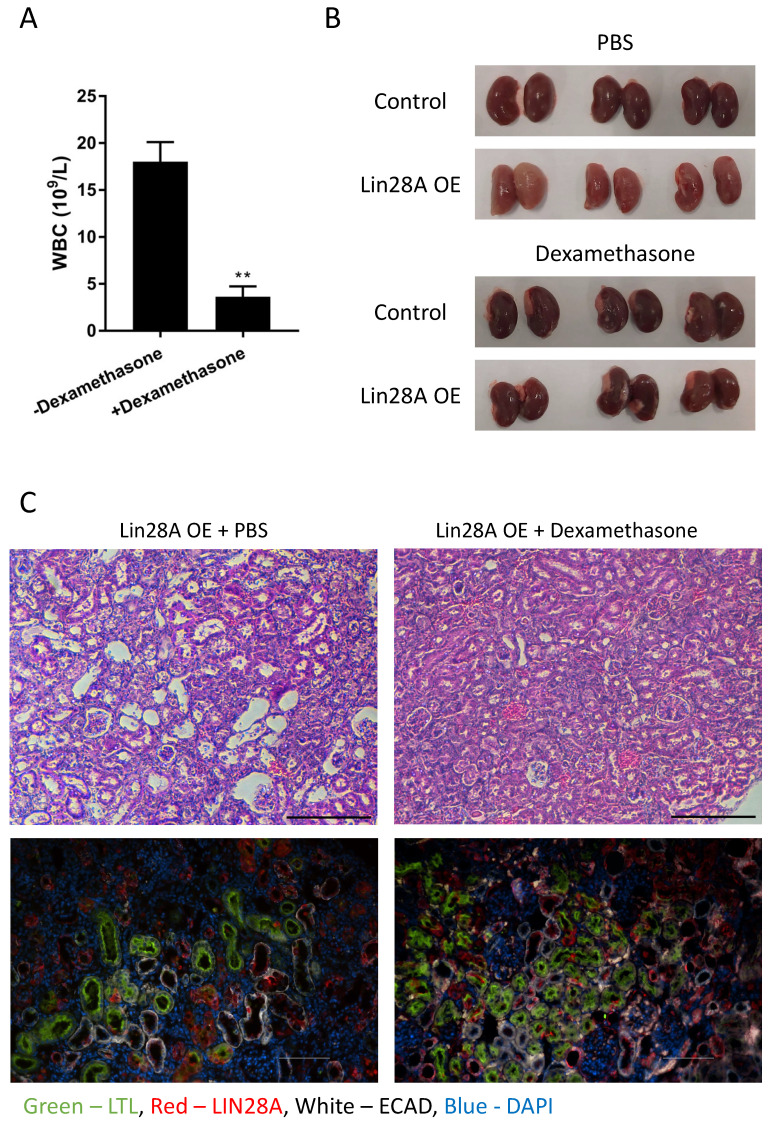
** Dexamethasone treatment prevents *Lin28A* OE-induced kidney damage. A.** White blood cell (WBC) count of *Lin28A* OE mice treated with dexamethasone/PBS for 3 weeks. **B.** Gross morphology of kidneys from *Lin28A* OE and control mice treated with dexamethasone/PBS for 3 weeks. **C.** H&E staining (upper panel) and immunostaining (lower panel) of *Lin28A* OE kidneys treated with PBS or with dexamethasone for 3 weeks. Kidneys were stained for LIN28A, PT (LTL), and DT/CD (ECAD). Scale bar - 100µM. Statistical analysis using Student's t-test. N=3. ** P<0.01.

To investigate whether the kidney damage results from the inflammatory response or if these are distinct consequences of *Lin28A* OE, we treated the mice with dexamethasone to prevent inflammation (**Figure 3A**). Remarkably, dexamethasone treatment completely rescued the kidney phenotype (**Figure 3B,C and Figure S4**). This result suggests that the *Lin28A*-induced inflammatory response caused the kidney damage. To explore whether the inflammatory response is induced when *Lin28A* is overexpressed in kidney cell types other than nephrons, we crossed the TRE-*Lin28A*;Lox-stop-Lox-TetOn-rtTA transgenic mice with *FoxD1*-Cre mice to induce *Lin28A* OE in the stromal cells of the kidneys^32^. While this crossing led to a significant *Lin28A* OE (**Figure S5A**), it did not cause an inflammatory response and kidney damage **Figure S5A,B)**. These results indicate that the effect of *Lin28A* OE on inflammation development is cell-context-dependent.

To determine whether *Lin28A* OE is required only for the initiation of the inflammatory response or also for its maintenance, we treated the mice with Dox for 2-3 weeks and then withdrew Dox for an additional three weeks. As expected, Dox withdrawal led to *Lin28A* downregulation (**Figure S6A**). This downregulation of *Lin28A* significantly decreased the inflammatory response, as evidenced by the downregulation of CXCL1, indicating that Lin28A OE plays a role in both initiating and sustaining the inflammatory response (**Figure S6B**). Remarkably, although the decrease in the inflammatory response did not lead to recovery of the kidney phenotype, it halted further progression of the damage (**Figure S6C**).

### The mTOR signaling pathway is upregulated but not involved in the kidney phenotype

**Figure 4:**
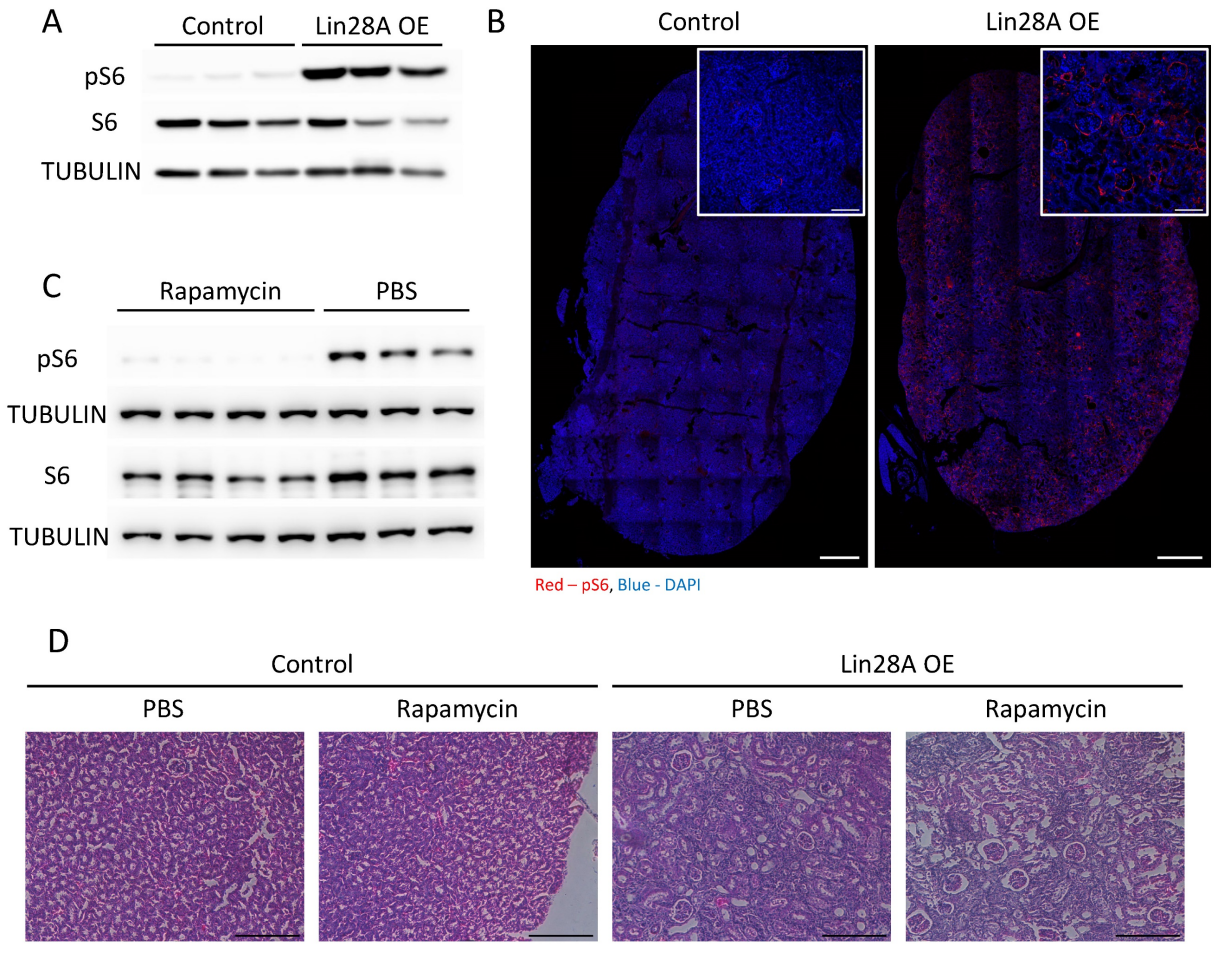
***Lin28A* OE leads to the activation of the mTOR pathway. A.** Western blot for phospho-S6 (pS6), S6, and αTUBULIN levels in control and *Lin28A* OE kidneys (3 weeks of doxycycline treatment). **B.** pS6 immunostaining in control and *Lin28A* OE kidneys. Scale bar - 500µM, inset scale bar - 100µM. **C.** Western blot for pS6, S6, and αTUBULIN levels in *Lin28A* OE kidneys upon rapamycin or PBS treatment. **D.** H&E staining of *Lin28A* OE kidneys upon rapamycin or PBS treatment. Scale bar - 100µM.

It has been shown previously that *Lin28A/B* regulate the activation of the mTOR pathway(11-13, which, among other functions, regulates the immune system response^33^ and whose abnormal activation has also been associated with kidney pathogenesis^34^. Indeed, we observed a significant activation of the mTOR pathway (as evidenced by increased pS6 levels) upon *Lin28A* OE in the kidneys (**Figure 4A,B**). Therefore, we treated the transgenic and control mice with rapamycin to explore if the mTOR pathway is also involved in the kidney phenotype induced by *Lin28A* OE. While the rapamycin treatment significantly inhibited the activation of mTOR pathway (**Figure 4C**), it did not rescue the kidney phenotype (**Figure 4D**). These results indicate that the effect of *Lin28A* OE on the kidneys is not mediated via mTOR pathway activation.

### The effect of *Lin28A* OE on the kidneys is mediated via the AKT-NF-κB-CXCL1 pathway

**Figure 5:**
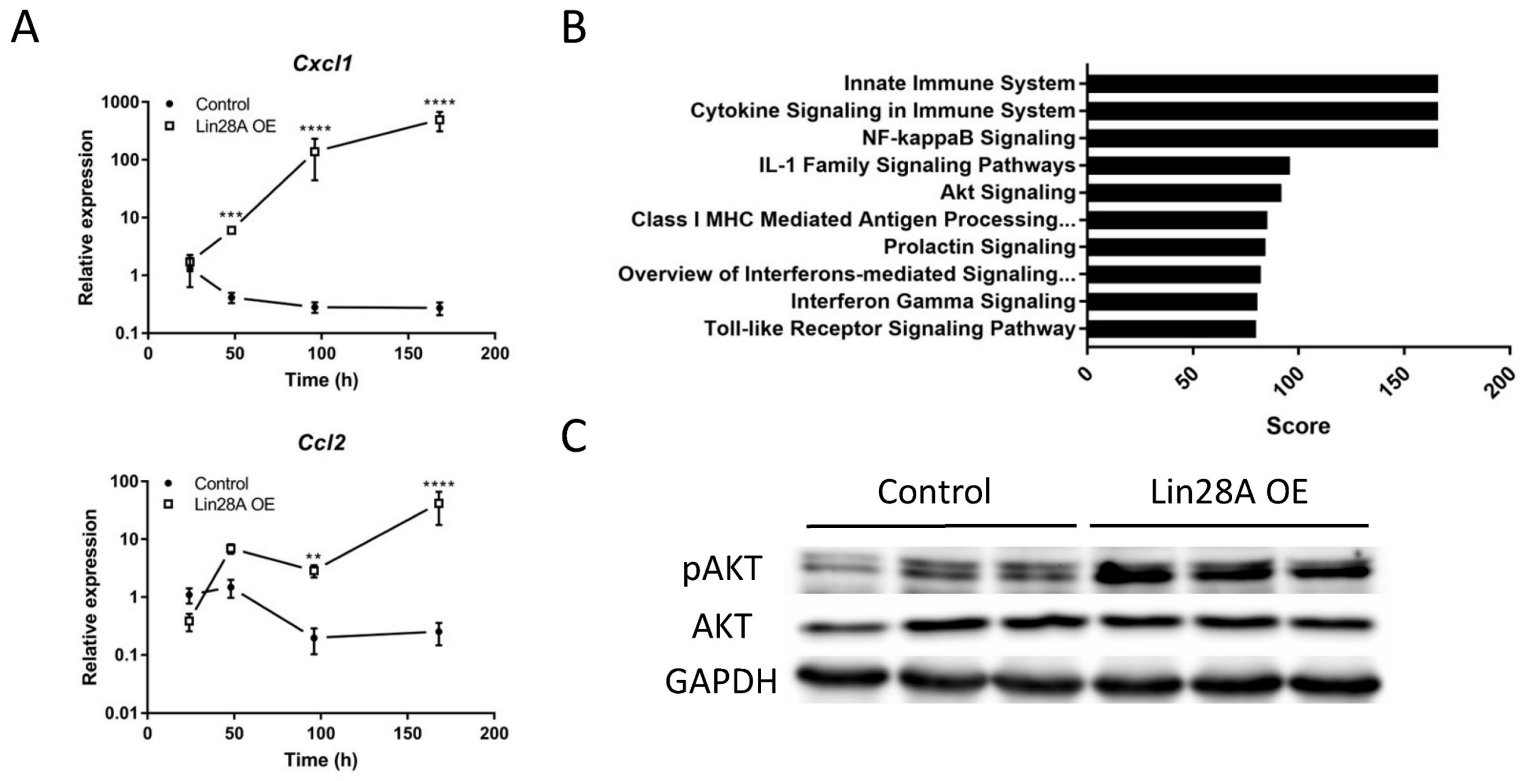
***Lin28A* OE-induced inflammatory response is mediated via the CXCL1-AKT-NF-κB pathway. A.** Time course analysis of *Lin28A* OE induced Cxcl1 and Ccl2 expression as determined by qRT-PCR. **B.** Top 10 enriched molecular pathways in *Lin28A* OE compared to control kidneys. **C.** Western blot for phospho-AKT (pAKT), AKT, and GAPDH levels in control and *Lin28A* OE kidneys following 48h of Dox treatment. Statistical analysis using 2-way ANOVA with Sidak post-test. N=3. **P<0.01, ***P<0.001, **** P<0.0001.

Our qRT-PCR and RNA seq data (**Figure 2D** and **Table S2**) reveal that *Cxcl1* is one of the most significantly upregulated cytokines upon *Lin28A* OE. We further validated this observation by analyzing the expression levels of *Cxcl1* in comparison to *Ccl2* at 24,48,96, and 168h following Dox treatment (**Figure 5A**). *Cxcl1* plays an important role in kidney inflammation and damage^35^,^36^ and is regulated by the NF-κB pathway^37^. Our RNA-seq results showed that the NF-κB pathway was one of the most significantly upregulated pathways in the transgenic kidneys (**Figure 5B**). NF-κB activation is controlled by the AKT pathway^38^, which has been shown to be upregulated upon *Lin28A* and *Lin28B* overexpression^13^,^39^. Indeed, we found that the AKT pathway was also upregulated in the *Lin28A* OE kidneys (**Figure 5B,C**). This upregulation takes place already about 24h following *Lin28A* OE (48h of Dox induction) (**Figure 5C**), indicating that this signaling pathway is a direct target of *Lin28A* OE in the transgenic kidneys. Together, these results suggest that the inflammatory response in the transgenic kidneys is mediated via the LIN28-AKT-NF-κB-CXCL1 pathway.

## Discussion

*Lin28A/B* are primarily expressed in stem and progenitor cells, play a crucial role in diverse cellular processes 7,8,11-18 and are overexpressed in various cancer types, acting as oncogenes (for reviews, see 5,22,24,40,41). In the current study, we show, for the first time, that *Lin28A* OE in mature nephrons results in a severe inflammatory response rather than in cell transformation. While we focused on *Lin28A*, our preliminary results (16 and data not shown) suggest that *Lin28B* OE in the nephron may have a similar effect on the kidney.

Whereas previous studies 28,42 have focused on the oncogenic implications of *Lin28B*-mediated inflammation in hematopoietic cancers, our research emphasizes the broader impact of *Lin28A (*and probably also *Lin28B)* dysregulation on inflammatory processes. We demonstrated that *Lin28A* OE in non-hematopoietic lineage can trigger an inflammatory response via the rapid upregulation of several cytokines, including CXCL1 and CCL2, leading to severe kidney damage. This response was not associated with tumor development and was mediated via IL-6-independent mechanism. It is worth noting that the mTOR pathway, which was upregulated in the kidneys of *Lin28A* OE mice, is known to play a role in tumorigenesis 43,44. This suggests that long-term activation of this pathway via Lin28A OE, while the inflammatory response is repressed (for example, by dexamethasone treatment), might eventually result in tumor formation.

In addition to our findings, several studies have demonstrated that the lipopolysaccharide (LPS)-induced inflammatory response is mediated, in some cases, through the upregulation of *Lin28A* 45-47. Although, in contrast to our results, the involvement of *Lin28A* upregulation in the inflammatory response was LPS-dependent and was not mediated via the Lin28A-AKT-NF-κB-CXCL1 pathway, these findings further illustrate the connection between *Lin28A* upregulation and inflammation.

Additionally, a recent study investigated the impact of high glucose treatment on human mesangial cells as a model to examine the influence of elevated glucose levels on the development of diabetic nephropathy 48. In this study, the authors found a possible link between *Lin28B* upregulation and secretion of IL-6 and TNFα. This *in-vitro* model is fundamentally different from our *Lin28A* OE *in-vivo* model. The differences include the specific *Lin28* isoform, the type of cells studied, and the cytokines secreted due to *Lin28A/B* expression. Yet, their findings provide another indication of Lin28's involvement in kidney inflammation.

To conclude, we show for the first time that *in-vivo Lin28A* OE in the nephrons can lead to a severe inflammatory response, likely via the Lin28A-AKT-NF-κB-CXCL1 pathway, independent of LPS or any other treatment. Our observation that *Lin28A* OE led to inflammation development only when overexpressed in the nephrons but not in the stromal cells of the kidney revealed that the effect of *Lin28A* OE is cell-context-dependent. However, the exact cellular conditions that lead to inflammation development due to *Lin28A* OE have yet to be determined.

One of the earliest cellular responses to acute kidney injury (AKI), which takes place within several hours of kidney damage, is neutrophil and macrophage infiltration^49^-^51^. This initial inflammatory response can then lead to necrosis of additional renal cells, thus enhancing the inflammation^30^,^52^,^53^. Therefore, we cannot rule out the possibility that the inflammatory response may be triggered by cellular damage induced by *Lin28A* OE, resulting in the secretion of damage-associated molecular patterns (DAMPs) 54. In this scenario, the AKT signaling pathway and its downstream effects might take place in immune cells recruited by the secreted DAMPs rather than in the nephron cells directly. However, we found that dexamethasone treatment not only prevented inflammation but also completely rescued the kidney phenotype. This finding along with the rapid activation of the AKT signaling pathway and the subsequent upregulation of *Cxcl1* and *Ccl2* levels within 24 hours following *Lin28A* upregulation strongly suggest that the inflammatory response is a direct effect of *Lin28A* OE.

Approximately 13.3 million patients per year are diagnosed with AKI 53,55. In addition to its acute effect, maladaptive repair of the initial kidney damage can lead to the transition of AKI to chronic kidney disorder (CKD) or worsening of baseline CKD 52,53,56. While several pathways, including inflammation, have been suggested to play a role in maladaptive AKI repair and progression of CKD, the pathophysiology of this transition is still not completely understood 52,57. Elucidating the molecular mechanisms underlying this transition will contribute to a deeper understanding of CKD pathogenesis and identify potential therapeutic targets for intervention. We demonstrated that three weeks of *Lin28A* OE followed by three weeks of its downregulation and cessation of the inflammatory response failed to recover the kidney phenotype but prevented it from becoming more severe. This observation suggests that our novel mouse model provides a valuable tool for investigating the conversion from adaptive to maladaptive repair and the development of CKD as a result of AKI. In a more general perspective, this *Lin28A* OE mouse model can be used to study other open questions regarding inflammatory kidney disorders, such as acute and chronic interstitial nephritis 58.

To summarize, our findings demonstrate that in specific cellular contexts, Lin28A OE can lead to a severe inflammatory response, resulting in significant kidney damage. These results suggest that the pathophysiological effects of Lin28A OE extend beyond cell transformation and raise the possibility that Lin28A/B OE could also play a role in human renal disorders associated with inflammatory responses.

## Supplementary Material

Supplementary figures and tables.
