# Supplementary material for: Nephron-Specific Lin28A Overexpression Triggers Severe Inflammatory Response and Kidney Damage
Source: Int J Biol Sci. 2024 Jul 22;20(10):4044–54. doi: 10.7150/ijbs.97434 (PMC11302891; doi:10.7150/ijbs.97434)
Supplement: Supplementary file 1 — Supplementary figures and tables. [file ijbsv20p4044s1.pdf]

SUPPLEMENTARY DATA

Figure S1

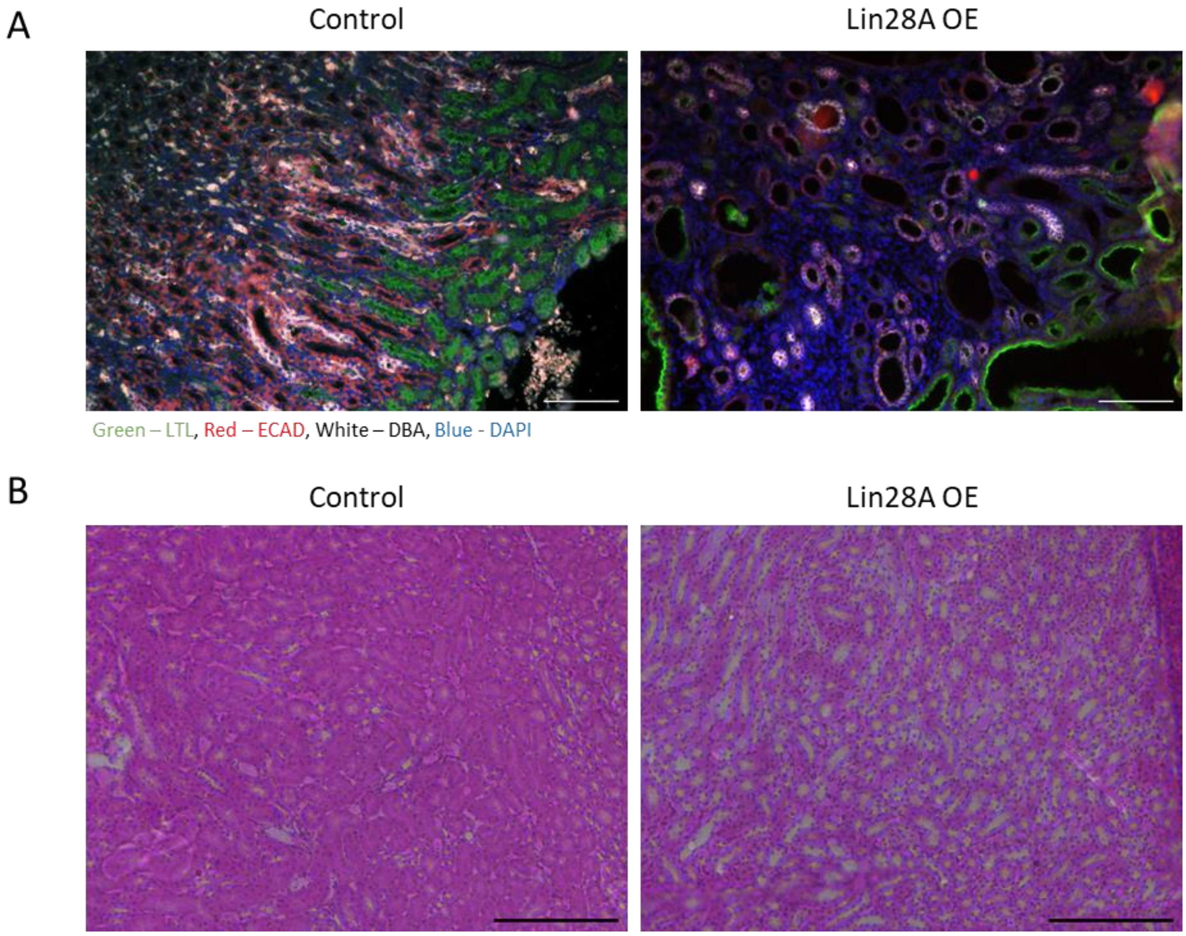

Figure S1. Characterization of the *Lin28A* OE-induced kidney damage. A. Control and Lin28A OE kidneys, following 5 weeks of Lin28A OE, stained for PT (LTL), DT (ECAD), and CD (ECAD, DBA). Scale bar - 100μM. **B.** H&E staining of control and *Lin28A* OE kidneys following 1 week of Lin28A OE induction. Scale bar - 100μM.

Figure S2

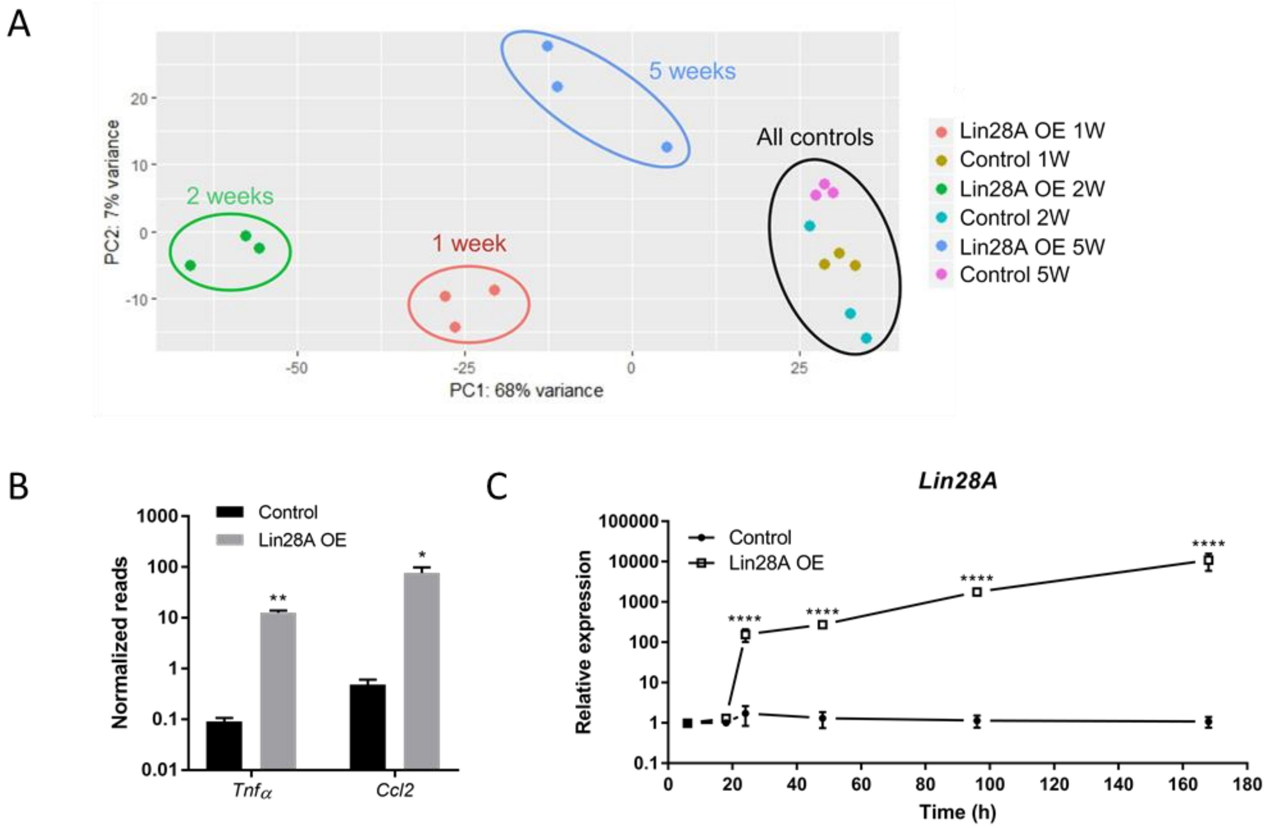

Figure S2. The effect of *Lin28A* OE on global transcriptome levels and specific gene expression. A. Principal Component Analysis (PCA) of the RNA-seq data, depicting the effects of different durations (1 week, 2 weeks, and 5 weeks) of Dox treatment on *Lin28A* OE compared to control mice. **B.** RNA seq normalized read counts of *Tnfα* and *Ccl2* in Lin28A OE kidneys compared to control kidneys upon 2 weeks of Dox treatment. **C.** Time course analysis using qRT-PCR to monitor the expression levels of *Lin28A* upon Dox treatment. Statistical analysis using Multiple t-tests with Holm-Sidak post-test (B) or 2-way ANOVA with Sidak post-test (C). N=3. * $P<0.05$, ** $P<0.01$, *** $P<0.001$, **** $P<0.0001$.

Figure S3

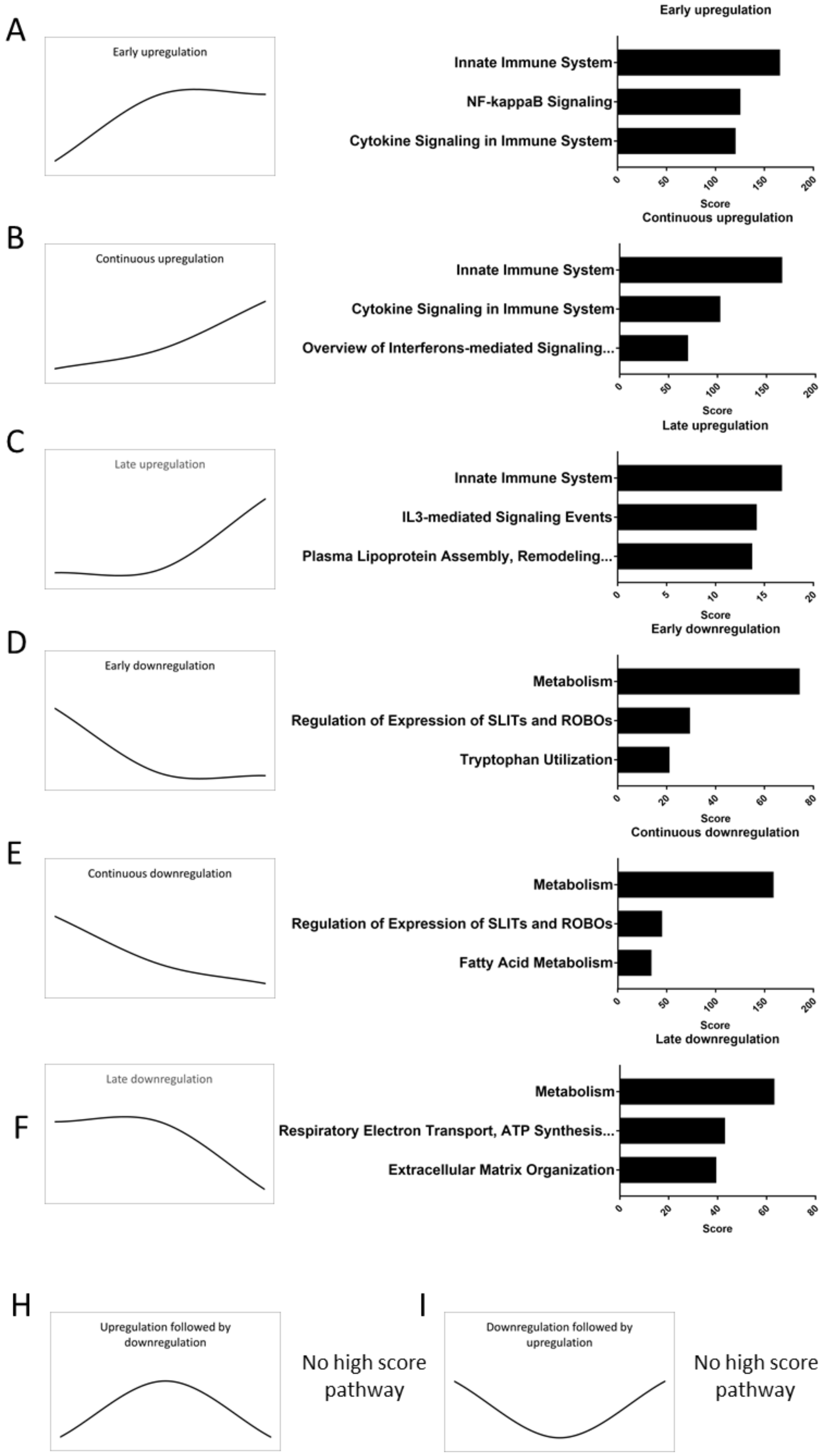

Figure S3. Analysis of gene expression dynamics over two weeks of *Lin28A* OE. Left and bottom panels – Illustration of the dynamic expression pattern for each category. Right panels - The most significantly enriched pathways in each category. Gene expression dynamics were categorized based on comparisons between 2W vs. 1W DEGs and 1W vs. control (0W) DEGs. N=3.

Figure S4

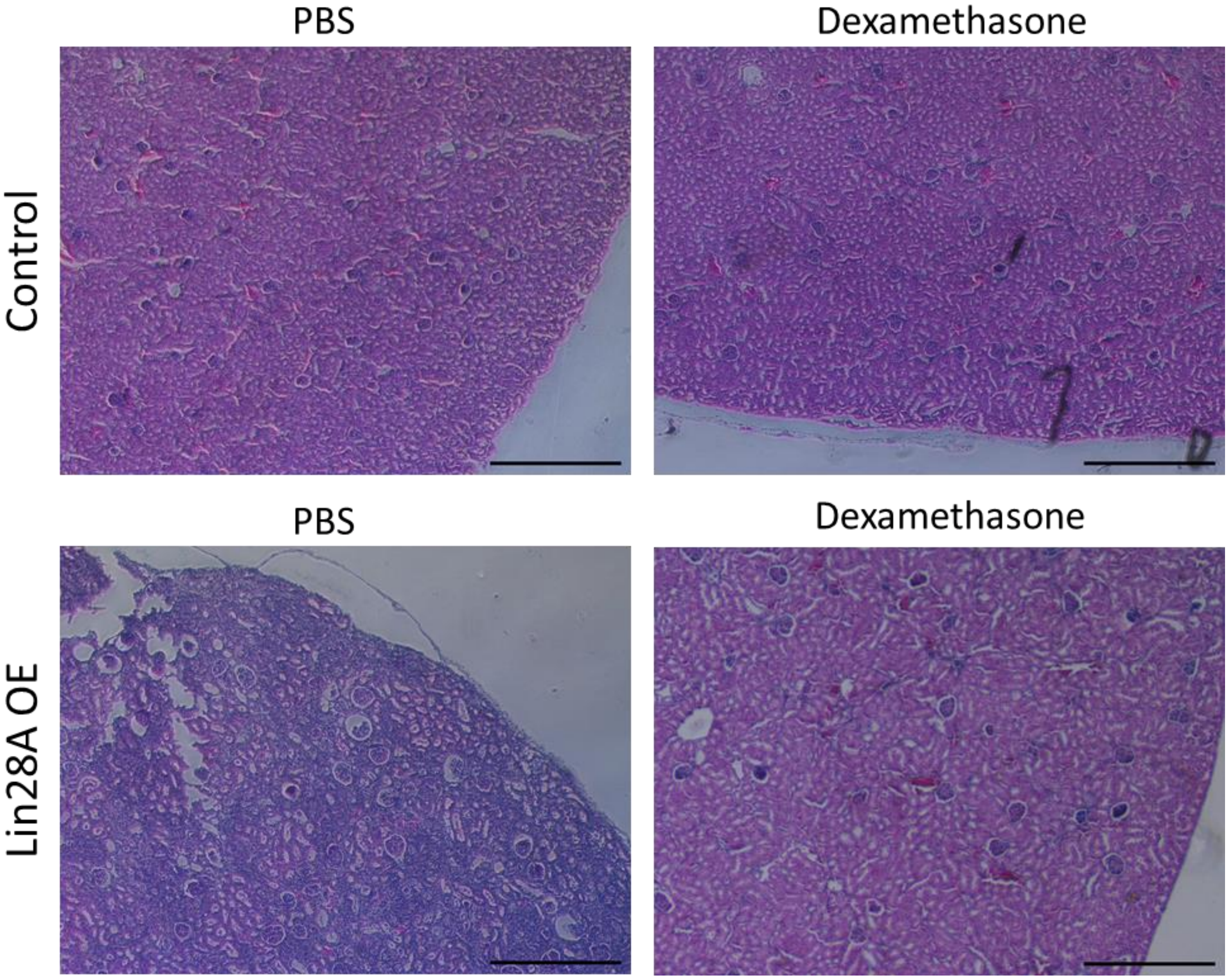

Figure S4 - Dexamethasone treatment prevents *Lin28A* OE-induced kidney damage. Representative images of H&E staining of kidneys from *Lin28A* OE and control mice treated with dexamethasone/PBS for 3 weeks. Scale bar - 500μM.

Figure S5

A

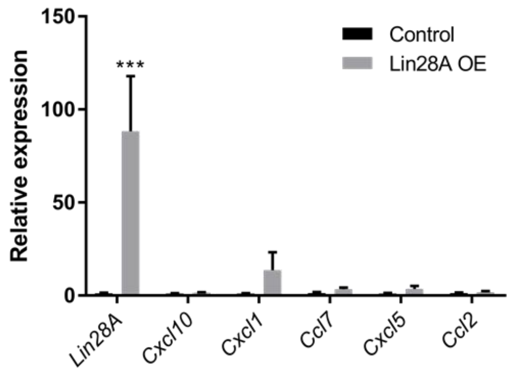

B

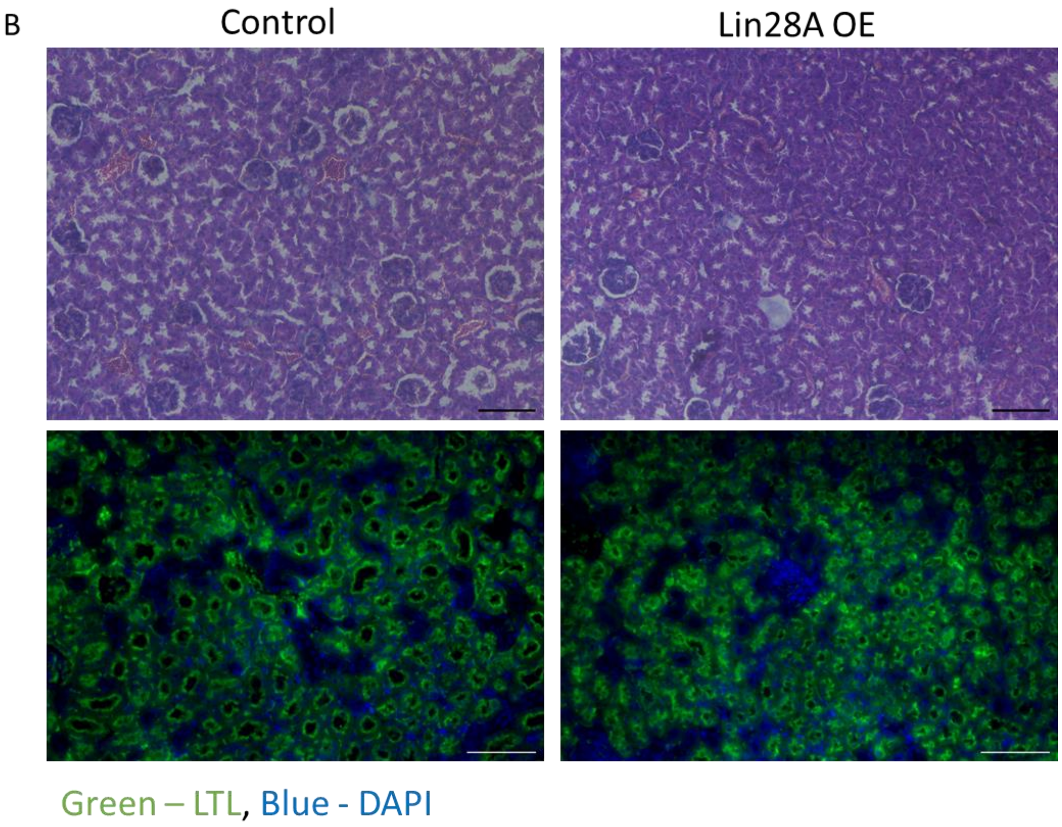

Figure S5. *Lin28A* OE in the kidney stromal cells does not induce an inflammatory response. A. qRT-PCR for *Lin28A* and several cytokines in kidneys derived from mice overexpressing Lin28A in the kidney stromal cells and from control mice. **B.** H&E staining (upper panel) and PT (LTL) immunostaining (lower panel) of kidneys derived from mice overexpressing Lin28A in the kidney stromal cells and from control mice. Scale bar - 100μM. Statistical analysis using Multiple t-tests with Holm-Sidak post-test. N=3-4. ***P<0.001.

Figure S6

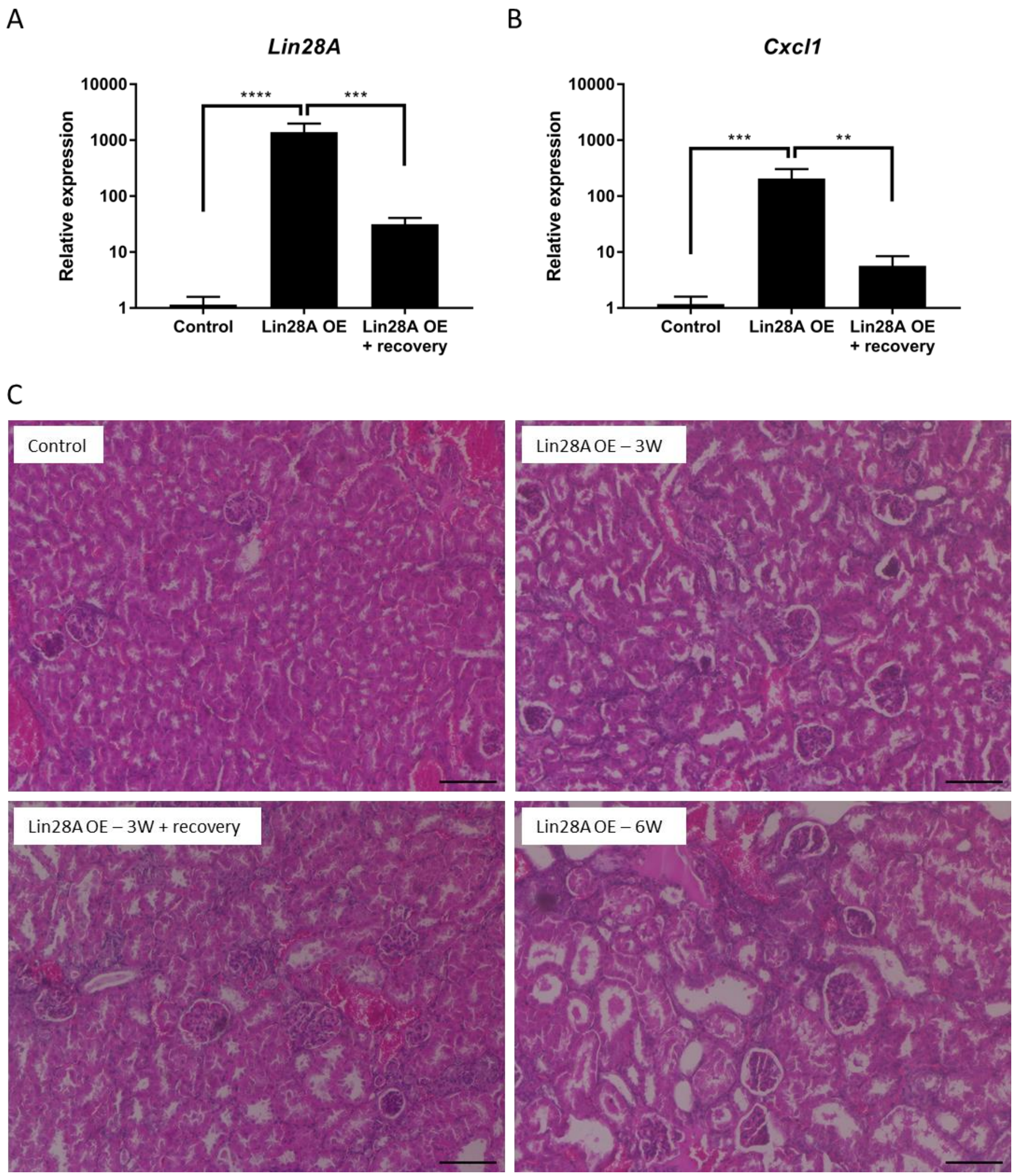

Figure S6. *Lin28A* downregulation attenuates the inflammatory response and prevents the worsening of kidney damage. A. qRT-PCR analysis of *Lin28A* and *Ccl2* levels in control kidneys, kidneys after three weeks of Dox induction (Lin28A OE - 3W), and kidneys after three weeks of Dox induction followed by three weeks of Dox withdrawal (*Lin28A* OE - 3W + recovery). **B.** Representative images of H&E staining of control kidneys, kidneys after Lin28A OE – 3W, Lin28A OE – 3W + recovery and Lin28A OE for 6 weeks (Lin28A OE - 6W). Note the increased severity of kidney damage after six weeks of Lin28A OE compared to the other conditions. Statistical analysis using one-way ANOVA with Sidak post-test. N=3 $^{**}P<0.01$, $^{***}P<0.001$, $^{**}P<0.0001$. Scale bar - 100μM.

Table S1. Antibodies and oligonucleotides used in the study.

Antibodies - Flow cytometry		
**Antibody**	**Source**	**Catalog #**
AF488-CD4 (1:800)	Biolegend	#100423
PE-CD8a (1:160)	Biogems	#10112-60
PerCP-Cy5.5-B220 (1:80)	Biogems	#07131-70-100
PE-Cy7-CD3e (1:200)	Biolegend	#100320
APC-CD45 (1:600)	Biogems	#0751280
FITC-MHC-II (1:330)	eBioscience	#11532185
PE-CD45 (1:500)	eBioscience	#12-0451-81
PE-Cy7- Ly6G (1:200)	BioLegend	#127618
PerCP-Cy5.5-Ly6C (1:80)	Biolegend	#128012
APC-CD11b (1:600)	eBioscience	#170112-81
APC-eFloure 780-CD11c (1:80)	eBioscience	#47-0114-80
**Antibodies and lectins - Western blot & immunostaining**		
Phospho-S6 Ribosomal Protein (Ser240/244) (D68F8) XP	Cell Signaling Technologies	#5364
S6 Ribosomal Protein (5G10)	Cell Signaling Technologies	#2217
LIN28A (D1A1A) XP	Cell Signaling Technologies	#8641
Akt (pan) (C67E7)	Cell Signaling Technologies	#4691
Phospho-Akt (Thr308) (C31E5E)	Cell Signaling Technologies	#2965
Alpha tubulin	DSHB	12G10
GAPDH	Millipore	ABS16
E-Cadherin	BD Biosciences	#610181
Keratin8	DSHB	TROMA-I
Dolichos biflorus agglutinin (DBA) - Rhodamine	Vector Laboratories	RL-1032-2
Lotus tetragonolobus lectin (LTL) - Biotinylated	Vector Laboratories	B-1325-2
**Oligonucleotides**		
	**Forward**	**Reverse**
CXCL1	ATCCAGAGCTTGAAGGTGTTG	GTCTGTCTTCTTTCTCCGTTACTT

CXCL5	ACAGTGCCCTACGGTGGAAGT	CGAGTGCATTCCGCTTAGCTT
CCL2	AGGTCCCTGTCATGCTTCTG	TCTGGACCCATTCCTTCTTG
CCL7	TGAAAACCCCAACTCCAAAG	CATTCCTTAGGCGTGACCAT
CXCL10	GACGGTCCGCTGCAACTG	CTTCCCTATGGCCCTCATTCT
CXCL2	CTCTCAAGGGCGGTCAAAAGTT	TCAGACAGCGAGGCACATCAGGT
CCL12	AGAATCACAAGCAGCCAGTGT	ATCCAAGTGGTTTATGGAATTCT
CCL20	CGACTGTTGCCTCTCGTACA	AGGAGGTTCACAGCCCTTTT
LIN28A	AGGCGGTGGAGTTCACCTTTAAGA	AGCTTGCATTCCTTGGCATGATGG
β ACTIN	AGCCATGTACGTAGCCATCC	CTCTCAGCTGTGGTGGTGAA
CXCL1	ATCCAGAGCTTGAAGGTGTTG	GTCTGTCTTCTTTCTCCGTTACTT

Table S2. Top 10 upregulated cytokines upon one week of Dox induction

Gene name	Log_2(fold change)	P adjusted
CXCL10	7.3	2.71E-130
CXCL1	8.2	3.07E-32
CXCL5	7.6	2.91E-15
CCL2	5.3	1.31E-14
CXCL2	6.8	5.38E-17
CCL7	6.1	4.45E-09
IL1F6	6.9	5.43E-15
TNF	5.2	4.10E-21
CCL20	9.0	4.71E-11
IL1F9	7.2	3.66E-13

Table S3 – DEGs representing gene expression dynamics.

Early upregulation	Early downregulation	Continuous upregulation	Continuous downregulation	Late upregulation	Late downregulation	Upregulation followed by downregulation	Downregulation followed by upregulation
erpinb9b	Gm10643	Gpnmb	2010204K13Rik	Sprr2f	Gm13341	Gbp6	Kif5a
BC023105	Aldh2	Cxcl5	Slc17a1	Crisp1	Serpinb8	Fcrls	Gm9752
Ccl20	1700001O22Rik	Serpinb9e	Hadh	Cyp2d9	Erlin1	Slc24a5	AI480526
Xist	Mamstr	Mylk2	Iyd	Ifi213	Scp2	Cfi	E230016M11Rik
Cxcl1	Dhdh	Serpinb9g	Acot11	Hal	Ccl21a	Lrrtm1	Nudt8
Cxcl10	Hemk1	Il1f6	Sdhb	Jaml	Mrpl50	Comp	4932422M17Rik
Il1f9	Whrn	Cxcl2	Abcc6	Muc3a	Gm26737	Fgl1	Ambp
Csf3	6430550D23Rik	Acod1	Tmem143	Gm21887	Sucla2	Gm15411	Mir5125
Rpsa-ps10	Lmod1	Gsdma	Fras1	Slc10a6	Slc47a1	Nxpe5	Tgfbr3l
Ccl7	Lmtk3	Gm4841	Hspd1	Rnf39	Fkbp4	Gm43697	Firre
Serpine1	Gphn	Apol9a	Fam107a	8430408G22Rik	Kcnab3	Dclk1	Klf15
Ankrd1	4430402I18Rik	Mx1	Zmynd10	Map3k6	Prom1	Slc13a1	Coro6
Tnnt3	Gcsh	Krt20	Slc28a1	Ifi206	Mapt	Mylpf	Ciart
Havcr1	Dusp23	Clec4d	Sod2	Igfbp1	Pomgnt2	Fst	AW822252
Ccl2	Coa6	Mmp7	Notum	Tnnt2	Chchd3	Fbln1	Fam163a
Cdkn1a	Dpf3	Oas3	Ush1c	Meltf	Tpmt	Thy1	Nat8
Zc3h12a	Abcb6	Oasl1	Isoc2a	Slpi	Gm43668	C430049B03Rik	Klk1b22
Nfkbid	Exoc3l2	Tnf	Gm19950	Lrg1	Amdhd2	Krt14	
Ifit1bl1	Gpt	Rgs1	Mpp4	Apoc3	Hdhd2	Foxl1	
H19	Ripply3	Fpr1	Icam2	Crabp2	Fh1	Penk	
Slc39a4	Zbtb20	Clec4e	Msra	Zbtb16	Arhgef9	C130021I20Rik	
Ms4a4c	Abca4	Oas1g	Atp2b2	Olfr920	Cox7a2	Mfap4	
Ccl12	4930506C21Rik	Ifit1	Nlrp6	Hap1	Ugt8a	Smoc2	
Cd300lb	Npr2	Fpr2	Nudt19	Gm11827	Slc6a18	Hmcn1	
Egr2	Sgk2	Lcn2	Slc33a1	Gm10800	Ccdc91	Ptgs2	
Fosb	Wwp1	Sprr2g	L2hgdh	Gm26797	Taco1		
Apod	Amt	Apol9b	Slc25a15	Abca6	Gm44764		
Il1rn	Tmem229a	Slc7a11	Glb1l	Slc43a1	Ddo		
Eda2r	Hnf1a	S100a8	Tcn2	Cd163	Atp5f1		
Serpina3f	Atp10d	Sirpb1c	Dmgdh	Plet1os	Idh3g		
Stx11	Mettl26	Gbp8	Chac2	Prkcg	Cycs		
Atf3	Slc22a4	Lif	Gjb2	Upp2	Prepl		
Gbp10	Gm16279	Ms4a6d	Gcdh	Slc17a9	Tmem260		
Eid3	Cpsf4l	Adam8	Egfl6	Fap	Slc25a19		
Tnfsf15	Gm28424	Gm5431	Mogat2	Gm15441	Gsta2		
Chac1	RP23-312B17.2	Edn1	Slc6a4	Fam124a	Idh2		
Serpina3g	Tbc1d16	Gm10364	Adk	Lingo4	Itga9		
Cyr61	Chadl	Cyp4f18	Hlf	Tbx3os1	Fam229b		
Cxcl11	Sycp3	Omp	Hhatl	Fcgr4	C330018D20Rik		
Timp1	Insig1	Gm10827	Aldh6a1	Galnt15	Mcoln3		
Gprc5a	Dpyd	Fosl1	Igsf11	Alox5	Zfp239		
Lect1	1810024B03Rik	Siglec1	Acad11	Adgrg2	Gm15848		
Lyz2	Sp5	Slfn1	Gm43667	Glt1d1	Prom2		
Mlf1	4833413G10Rik	Rnd1	Gpr155	Gm38309	Atp5h		
Tgtp1	Evpl	Ifit3b	Adtrp	Pik3ip1	Podxl2		
2310043M15Rik	Rad9b	Psors1c2	Slc5a11	Pydc3	Lama1		
Cxcl9	Tenm4	Ms4a6c	Rasgrp3	Olfr1372-ps1	Svip		
Wnt10a	Gsta3	Fgb	Aldh7a1	Gm15222	Lmbrd2		
Ccl5	Nsdhl	Isg15	Lpl	Hp	Fads2		
Hhipl2	Rapsn	Mx2	Csad	Gm45221	Supt3		
Clec12a	Nadsyn1	Angptl8	Etfa	Gm20605	Gimap8		
4930539E08Rik	Mmp15	S100a9	Fahd1	Gm15708	Napsa		
Clec4n	Clybl	Hcar2	Cth	Rprm	Lrpprc		
Cd44	Fhit	Zbp1	Pepd	Apoe	Adgrl3		
Gadd45b	Fgf9	Adgrg7	Insrr	Tmem240	Robo1		
Ms4a6b	2310039H08Rik	Slfn4	Tmem174	Bglap3	Echdc1		
Csf1	Phlda2	Trib3	Ppp1r16b	Chn1os3	mt-Nd5		
Igf2bp2	Sirt3	Chil3	Lrpap1	Pigr	Cyb5rl		
Ccl8	Slc12a6	Oas1a	Meox1	Pde1b	4930461G14Rik		
Il1b	Prcp	Oas2	Ksr2	Gm20033	Gm11837		
Tnfrsf12a	Gm15217	Fgg	Apeh	4933439C10Rik	Ccdc8		
Lgi2	Nat2	Gstp2	Gm37336	Gm43196	Rab6b		
Plekhs1	Lypla1	Cxcr2	Stxbp5l	B230303O12Rik	Ecm2		
Slc34a2	Lyrm4	Serpina3n	Hgd	Epop	Emilin1		
Afp	Efr3b	Itgam	Tcea3	Cyp2a5	Thrb		
AI506816	Ppox	Irf7	Acaa1b	Upk3bl	Ift80		
Ms4a4a	Echdc2	Cd84	Fbln7	Col11a2	Phkb		
Slc6a19os	Gm11766	Ccl9	Dnajc12	Mt1	Mfap3l		
Chil1	1810034E14Rik	Iigp1	Cat	Ppfia4	Hikeshi		
Igf2	Selenbp1	Tlr8	Gstk1	Gm973	Gm20939		
Adamts4	Perm1	Oasl2	Sardh	Msln	Pdp2		

Tor3a	Ccdc6	Msr1	Cd300lg	Mnda	Zfp366		
Ms4a7	Syn2	Ifit3	Ptprd	Epb41l4aos	Clcn5		
Olfr56	Sms	Vgf	Abhd3	Prkg2	Add3		
Ubd	Celsr2	Bdkrb2	Tet1	Gm20219	Aifm1		
Aif1	Ankrd24	Ctss	Dao	Neb	B4galnt3		
Parp14	Abhd14b	Clec5a	C1qtnf3	Pla2g7	Robo2		
Serpina10	Zfp692	Muc4	Slc22a18	Fkbp5	Hs3st3a1		
Hif3a	Abcc2	Ifit2	Bdh2	Gm10382	Uqcrc2		
Pvr	Rnft2	Lyz1	2500002B13Rik	Ttc9	Sult1b1		
Ncf4	Skida1	Fcgr1	Camk4	Tcf23	Trpv5		
Flnc	Per3	Asprv1	Dhfr	Id1	Tnfaip8		
Ccl28	Rfng	Usp18	Smim24	Calcr	Ndufa1		
Hk3	Vldlr	Ccr1	Apob	Gm43300	Fbp2		
Bcl3	Slc22a17	Gbp3	Cmbl	Dnase1l2	Nr0b2		
Gdf15	Gm36936	Trim6	Slc27a2	Klhl6	Arhgef15		
Stra6l	Abhd18	Rnf223	Kynu	Tmem40	Wnk2		
Plcxd2	Sod3	Il1r2	Qdpr	Gm12992	Prox1		
Cd4	Gm43031	Csf3r	Slc18a1	Aldh3a1	Decr1		
Rgs16	Tysnd1	Igsf6	Ces1e	A730011C13Rik	Ggt6		
Ifi211	Cit	BE692007	Mthfd1	Rbm46	Tcf21		
Gm15895	1500026H17Rik	Art5	Acadm	Trim14	Dusp9		
Gm4951	Ushbp1	Gpr141	Slc16a2	Mfsd2b	Idh1		
Icam1	1600014C10Rik	Ifi205	Retsat	Nanos1	Zfp607b		
Plaur	Rhcg	Ifi204	Aldob	Gm17251	Pank1		
Hmox1	Metap1d	Adam23	Amacr	B230208H11Rik	Pctp		
Hesx1	St8sia6	Havcr2	Cideb	Gm44103	Uqcr11		
Ltb	Csrp2	Casp4	Cgref1	Txnip	D630003M21Rik		
Fos	Bcl11b	Dusp5	Proc	B3gnt3	Tpk1		
Smim3	Cyp1a1	Acsbg1	Esr1	Trim5	Asl		
Serpina3i	Acbd4	Mpeg1	Papss2	Gm20632	Atp2a3		
Igtp	Myo7b	Nlrc4	Ephx2	Stk32c	Cox6c		
Gm28809	Aldh5a1	Gbp2	Snx29	Col13a1	Tsga10		
Gm13212	Atp6v1c2	Ifi207	Npy6r	Gbp4	Bckdhb		
Ly6c2	BC049762	Tnfaip3	Lrrc66	Ppp1r15a	Gm13910		
Il20rb	Tef	P2ry13	Slc25a21	Inha	Gm4430		
Trem2	Marcksl1-ps4	Slc2a6	Bpnt1	Npff	Col16a1		
Il18rap	Slc5a10	C1qa	Ttc36	Cldn23	Zmat1		
Ccdc109b	Ces2c	Arl11	Slc16a9	Mlph	Nme5		
Socs3	1700040L02Rik	Ifi209	Acsm5	Mmp9	Prrx1		
Cd52	Grtp1	C1qc	Wdr93	1700109H08Rik	Kif21a		
C1qb	Fmo4	Rtp4	Aqp11	Tfap2a	Pclo		
Ier3	Nrarp	5430430B14Rik	Acaa2	Per1	Fzd1		
Gm10557	Erc2	Wnt9a	Tkfc	Leng8	Gm17750		
Lair1	Sema4g	Ifi47	Car12	Uba7	Cdkl1		
Ccr5	Coasy	Trim30b	Gamt	Abcg1	Pantr1		
Tas1r3	Abca3	Mirt1	Mettl7b	Smpd5	Adra1a		
Adgre1	Slc25a10	Slc7a3	Cacna2d2	Clca3a1	Tnc		
Nfkbie	Adrb2	Rsad2	Acy3	Dbndd1	Eml6		
Gpr65	Chpt1	Klf6	BC025446	Aoc2	Tenm3		
Clec4a1	Fads1	Klra2	Slc7a8	A930001C03Rik	Akr1b7		
Pik3r5	Ttc39c	Cd68	Arsb	B230206H07Rik	Dkk2		
Glipr2	Slc46a1	Serpina3e-ps	Susd2	Itgb3	Sulf1		
Cd300c2	Gm35339	Gzmm	Rtn4ip1	Ppl	Tfec		
Vcam1	Vwa8	Atp1a3	Map3k7cl	2900005J15Rik	Tmc5		
Lhfpl2	Timp3	Slfn8	Scrn2	Clu	Eml1		
Egr1	Guca2b	C3ar1	Khk	C130071C03Rik	Ldb2		
Rnd3	Vps8	Ncf1	Eci3	Clca3a2	Gpx6		
Timd2	Slc51b	Rin1	Aifm3	5031434O11Rik	Map1a		
Hck	Gm16437	Aim2	Fam213b	Aldh1a1	Cox7b		
Irf4	Trpc1	Cd300lf	Mccc2	4930581F22Rik	Wdpcp		
Tlr2	Aco1	Oas1b	Erich4	C230096K16Rik	Hacl1		
Lcp2	Gm43980	Nlrc5	Fbxo40	1700029I15Rik	Thap2		
Fgd3	Ivd	Gbp7	Gpd1	Tacstd2	Spc25		
Hpgds	Vwa2	Cldn4	Fam217a	Gm21781	Gm11695		
Pmaip1	Aoah	Junb	Pbld2	Noxo1	Isyna1		
Dtx4	1810059H22Rik	Retnlg	Ryr2	Ifitm10	Ntrk1		
Il11	Gm5678	Psd4	Gm5424	Crtam	Ccdc171		
Clec4a3	Gstz1	Nefm	Me1	Gm44829	Slc22a1		
Gm12250	Ubxn10	Mlkl	Pth1r	Rbp4	Gprasp2		
Serpinb6b	Pln	Lilrb4a	Enpp3	Lox	Htr2b		
2510016D11Rik	Gm45051	Csf2rb	Sptlc3	Klf4	Acyp1		
Clec4a2	Pappa2	Il34	C030005K06Rik	Tsc22d3	Slc2a12		
Ifrd1	Nipsnap1	Cp	Vwa1	Gtpbp2	P4ha1		
Arntl	Fhl1	2200002D01Rik	Ace	Ltc4s	Esrrg		
Irgm2	Nr1h4	Tlr13	Apln	Hrct1	Lpar4		
Ccl11	Gm45453	C3	Aldh8a1	S100a14	Me3		

Aldh1a2	Frem2	Map3k8	Xylb	A530020G20Rik	Flrt3		
RP23-187B11.11	Mme	Dtx3l	Emx1	Ly6e	Itgb3bp		
Nos2	Slc25a42	Cd274	Nnt	Liph	Clic3		
Ddias	Dnhd1	Lgals3	Acot13	Slc25a33	Itga1		
Maff	Tmem88b	Arl4c	Pcdh17	Capn5	Eaf2		
Csf2rb2	Gpr17	Dhx58	Dnajc6	Slc25a22	Ccpg1os		
Dapp1	Klf1	Dpep2	Alpl	Slco2a1	Ikbip		
Cx3cr1	Acox3	Fga	Slc2a5	Whamm	Kcnd3		
Epsti1	Kcp	Tmem252	Hnf4aos	Tmem71	Magi2		
Spp1	C8g	Psd	Entpd8	Gda	Fbxo2		
AI839979	Myom3	Sla	4632428C04Rik	Adgrg3	Mfsd4b1		
Zfp568	Pxmp2	Cybb	Galnt14	Gm17494	Suclg2		
Hbegf	BC026585	Fcgr3	Slc5a8	Gabre	Nxph3		
Smpdl3b	4732463B04Rik	Ddit3	Adhfe1	Gm12319	Slco4c1		
Ly86	Robo3	S100a6	Anpep	1600020E01Rik	Npnt		
Nfkb2	C330002G04Rik	Gbp5	Fgfr4	1700102P08Rik	Ltbp1		
Fam212b	Nr1d1	Slc7a5	Calml4	Gm26917	Inpp4b		
Tyrobp	Cda	Plin2	Acss2	C920006O11Rik	Usmg5		
RP23-468H22.7	Gm44005	Casp1	0610005C13Rik	Klc3	Gper1		
Clec7a	Abcb1b	Slc11a1	D630023F18Rik	1810010H24Rik	Stxbp4		
Rtn4rl2	Fam57b	Ccdc88b	Espn	Tnk1	Sema3d		
Alox5ap	Ascc1	Btg2	Acsm1	Itgb4	Slc7a9		
Lax1	2610528J11Rik	Mt2	Hbb-bs	Ccrl2	Gm16861		
Dcdc2a	Tst	Rnf180	Cbs	2700099C18Rik	Hsph1		
Ptpn7	Defb29	Lgals3bp	Crocc	Arhgap45	Fbn1		
Amica1	Sfxn5	Slfn2	Galnt11	Avil	Ppp1r14a		
Pik3ap1	Tmem26	Bst2	Dleu7	Gm38366	Gja5		
Glipr1	1810017P11Rik	Scn3a	Lgr6	Doc2g	Ank3		
Rac2	Ccnd1	Pirb	Mut	Wdr62	St6galnac3		
Plk2	Pld6	C5ar1	Aldh4a1	Phkg1	Syt17		
Tnfrsf1b	Myh14	Tap1	Glyat	Scg5	Afg1l		
Aoc1	Akr7a5	Psmb8	Gm6652	Arhgap22	Fgf1		
Rassf1	6430511E19Rik	Hmga1	Lrrc19	Tcirg1	Rgs9bp		
Gpr34	Wscd2	Stat2	Fggy	Gm47283	Aco2		
C7	Treh	Cxcl13	Kmo	Slc25a47	D330045A20Rik		
Ahnak2	2810030D12Rik	Xaf1	Oxgr1	AW011738	Gm43618		
Pld4	Pde4c	Tmem154	Lpar3	Gm42798	Pcdh19		
Ifi27l2a	Cacnb4	Pak3	Abat	Pacsin3	Ak7		
Irgm1	Rtp3	Hilpda	Slc4a1	Islr2	Nusap1		
Rrad	Acad10	Runx3	S100g	Gm43860	Gsta4		
Tnip1	Kcnn2	Ifih1	Gcat	Creb5	Tmem14a		
Gm16685	Hebp1	Klf5	B3galnt1	D930048N14Rik	Plcb1		
Tnfsf13b	Gm17249	Herc6	Angptl7	Ctsd	Epha7		
Tspan32	Gm13262	Cd33	Gm42697	Tnfsf10	Slc16a10		
Milr1	Gm10658	Parp12	Apom	Slc25a27	Ptgds		
6820431F20Rik	Gm45170	Runx1	Cpb2	Gm44949	Sfxn2		
Rian	Kdr	Oit1	Pcsk9	Ovol2	Sema3b		
Lst1	Adssl1	Pglyrp1	Agxt2	Mapk11	Atp5k		
Lat2	Ar	Krt18	Tln2	Ttll11	Tek		
Nr6a1	Dhrs4	Ptprh	Slc26a4	Fam46b	Slc16a13		
Amotl2	Pipox	Tlr7	Pdzd3	N4bp2l1	Ptpro		
Lpcat2	Hnf1aos1	Nfkbia	Tinag	Ctxn1	Reln		
Mrc1	Map3k15	Trim30d	Slc8a1	Mir22hg	Slc4a11		
Cd53	Lrrc31	Sh2d5	Ces2e	Dgkh	Cspg4		
5730559C18Rik	Nit2	H2-K1	Ggt1	Csf2ra	Elovl6		
Ptpn12	Esm1	Anxa1	Agmat	Acrbp	Abcg2		
Tnfaip8l2	Grip2	Ddx60	Abhd14a	Klk1b3	Sfrp2		
Cd48	Adamts15	Trim30a	Idi1	Irs2	Gria4		
Serpina3h	Fam195a	Cldn3	Glyctk	1500011B03Rik	Cpt2		
Vav1	Magix	3010003L21Rik	Bphl	Fcgr2b	Hibch		
Snx20	Slc37a4	Nipal1	Car14	Gm6545	Stk32b		
Osmr	Gm38102	Nlrp3	Sord	Card9	Slc2a10		
Ms4a4b	Mgll	H2-T10	Lctl	2900089D17Rik	F8		
Dyrk3	Gm12121	Fut2	Slc16a7	Amigo3	Trib2		
Ajuba	Fah	Snhg15	Acat1	Gm13689	Sel1l3		
Il21r	Ceacam2	Basp1	Ass1	1810032O08Rik	Alx1		
Serpinb9	Dcxr	Zfas1	Lrrk2	Cxcr4	Nhlrc4		
Atg9b	Gldc	Naip5	Slc51a	Gm26532	Ndufa4		
Lrrc25	Gpr135	Il10ra	Acot4	Snhg17	Fam167b		
Cfap157	Reps2	Arid5a	Gm3716	Gm26836	Trpc3		
Atp8b4	Pck1	Pstpip1	Enpp6	A630001G21Rik	Col14a1		
Relb	Gm37844	Fgr	Gjb6	AU022754	Dusp19		
Fyb	Lhx1	Cldn7	Rida	Nbl1	Padi2		
Arid3b	1700067K01Rik	Tgif1	Gm4131	Trim34a	1700101I11Rik		
Misp	Capn6	Anxa3	Mgam	Tmem198	Hspa12b		
Cygb	Frs3	Cyp3a13	3632451O06Rik	Cabp1	Klhl14		

Plac8	Cisd3	Cmpk2	Calb1	Rasa4	Senp8		
Plagl1	Aldh9a1	F630028O10Rik	Gys2	Phf21b	Clstn2		
Plek	Lockd	B4galnt1	D630029K05Rik	Pkd1l3	Mrc2		
Gm42793	Gm11611	H2-Q4	Thnsl2	Gm13375	Hs3st6		
Samd5	Tmem25	Ddit4	Hykk	Kcnd1	Olfml3		
Was	Cyb5a	Akap12	Paqr9	Lmbr1l	B230369F24Rik		
Slc15a3	Ghr	Mthfd2	Actn3	Ankrd16	Ndufc1		
Evi2a	Dnajc19	Il18bp	Hcar1	Six2	Tyw3		
Adamts1	Ggnbp1	Socs2	Gm44386	Wfdc2	Gpr153		
Siglece	Fasn	Smox	Pcsk6	Arg2	Fam162a		
Zfp52	Ovol1	Spsb1	Serpinf2	1810026B05Rik	Chst2		
Neurl1a	Gm14257	H2-D1	Arhgef39	Coro2a	Htr1b		
Coro1a	Tmem150a	Nfil3	Smlr1	4933407K13Rik	A330009N23Rik		
Clcf1	Slc26a10	Soat2	0610006L08Rik	Gm4673	Tbxa2r		
Gm527	Gm36028	9930111J21Rik2	Gk	5330438D12Rik	Arhgap24		
Cfp	Wnt9b	Nr4a1	A1cf	Xrcc3	Vil1		
Myo1g	Shmt2	Rhou	Nap1l5	Pnp2	Uros		
Socs1	Dlec1	Eif4ebp1	Hdhd3	Hoxb5	Dchs1		
Map3k14	D7Ertd443e	Rnf19b	Lyplal1	Gch1	Plxna4		
Abl2	RP23-462D5.5	Sox9	Fbp1	Gnrh1	Atp5g1		
Myo1f	Lhx1os	Ercc1	St8sia1	Cd37	D630023O14Rik		
Atp2c2	Tmem64	Spred3	Acy1	Fxyd4	mt-Tp		
Mettl6	Vill	2410006H16Rik	Cyp51	Gm32856	Aspm		
Plet1	Mlxipl	Tmprss4	Pcca	Grn	Sdha		
Pilra	Pdzd2	Ctgf	D3Ertd751e	Galnt6	Enpp2		
Gm10226	Sap30	AW112010	Nox4	Gm15832	Pfkm		
Nckap1l	Mvd	Il18r1	Slc23a1	Cnr2	Hnmt		
Nek6	Acp6	Lrrc20	Gcnt1	Fchsd1	Wdr78		
Trim12c	Gm31105	Tnfrsf23	Ube2u	Klf8	Sertad4		
Lsp1	Pyroxd2	Ifi44	Pter	Rps4l	Slc13a4		
Sash3	Tmem86a	Sell	Pecr	Ago4	Bmp7		
Prrg4	Hmcn2	Hmga2	Macrod2	Blnk	Slc35f1		
Dusp8	9030617O03Rik	Gbp9	Gpm6a	Ethe1	Srl		
Birc3	Dpep1	Irak3	Nat8f1	Fbxo32	Slc17a4		
Laptm5	Gchfr	Phf11d	Slc22a13	Snhg5	Pgm5		
Parp9	Gm44243	Eno2	Lactb2	Gm12098	Ak1		
Car13	Ugt1a7c	Bbc3	Cmah	Fam46a	Scube1		
Zc3hav1	4833439L19Rik	Vat1	Pfn3	Gm43737	Isoc2b		
S100a4	Ano4	Meg3	Fn3k	Gm13423	Edn3		
S100a10	Aacs	Ifnlr1	Lin7a	Zc3h6	Nxnl1		
Vps37b	RP24-94F12.6	Abca1	Alas2	Hspb1	Col4a3		
Enkur	Hsd3b3	Snx10	Slc22a2	Cnksr1	Hnf4a		
Gm9951	Fam169b	Etv4	Slc22a6	Cbarp	Armcx4		
AB124611	Atp11a	Parp10	Chrna4	Bhlhe41	Kcnj5		
Tgtp2	RP23-306P12.3	Cebpd	Dio1	Fnbp4	Gatb		
Fcer1g	Rhbg	Slc6a9	L3hypdh	Gm15545	Gcgr		
Nup62cl	Cpeb3	Gcnt4	Tlr12	Malat1	Tspan2		
Tlr1	Chrm3	Xdh	B3galt5	Dnah17	Car8		
Dusp2	Col27a1	Adamtsl4	Gm10222	Gm11772	mt-Cytb		
Serpinc1	Tmem182	Ager	Them7	Ubc	Zfp354b		
Fbxo30	Gm16170	P2rx7	Cubn	Nrbp2	D10Jhu81e		
Trpv2	9430037G07Rik	Dok3	Gm13855	Jade2	Ank2		
Asb5	Gm15241	Plekhn1	Slc16a4	Gm17491	Gm2415		
Apobr	Pex5l	Daxx	4931406C07Rik	Shisa5	Rab11fip3		
Golm1	Matn4	Gpr161	Sypl2	Gm6977	B130034C11Rik		
Ciita	Paqr7	Tapbp	Ugt2b34	Cebpb	Zfp493		
Zfhx4	Adm2	Gm42636	Tmigd1	Spire2	Cdhr2		
Mgp	Atxn7l2	Ccdc9	Acsm2	Edaradd	Clmp		
Phlda3	Ren1	Trafd1	Cox7a1	Gm14137	2310007B03Rik		
Sh2d4b	Gm15879	Isg20	Clstn3	Gm43534	D430019H16Rik		
P2ry12	Pax9	Lgals9	Nat8f6	Cited4	Npb		
Zfp51	Sspo	Gm9442	Wfdc15b	H2-T23	Arxes2		
Samsn1	Dcst1		Rflna	Qtrt1	Prps2		
Npas2	Osbp2		Oxct1	Nmb	Plek2		
Gna15	G6pc		Acox2	Tap2	Frem1		
Arhgap30	Tsku		Slc13a3	Znfx1	Bsnd		
Zcchc18	Acot12		Akr1d1	Mapk13	Fabp4		
Dock2	Reep6		Nat8f3	Rpl10a-ps1	Sytl4		
Spi1	Vstm2b		Afm	Gm37422	Col5a1		
Sfn	Gm43481		Cckar	Pstpip2	1700028J19Rik		
Cd86	9630013D21Rik		Mep1a	Clasrp	Kif11		
Tgfbi	Dbp		Proz	Gm43197	Chid1		
Enc1	Fabp7		AI314278	Triobp	Fads6		
Pvt1	Pcsk4		B3gat2	Cirbp	Stard4		
Klhl30	Gria3		Gm15823	Vars	Nme4		
Apbb1ip	Mfsd2a		Hba-a2	Sh3bgrl3	Entpd5		

Ikzf1	Lipg		Hbb-bt	Slc2a8	Raver2		
Apobec1	F5		Gm15638	Pitpnm1	Il27ra		
Wnt16	AI838599		Gm15962	Krtcap3	Suclg1		
Pik3cg	Dhtkd1		Dusp15	4930563D23Rik	Dapk2		
Itgal	Gm10804		Olfm3	Bmp3	St6galnac5		
Zfp202	D230017M19Rik		Slc5a2	Arrdc2	Tspan18		
Tmem173	Gm43190		Mpv17l	Adra2a	Fbln5		
Trp53inp1	Spag8		Gas2	Slc37a1	C1rl		
Casp12	Apoa2		Aspa	Ifit1bl2	Spsb4		
Hcn3	Cyp2c44		Gm4524	Vsig2	Stmn1		
Selplg	Afmid		Cela1	Snhg20	Gm27202		
Adgra1	Gm26588		Sucnr1	Rps6kl1	Negr1		
Snhg1	Ugt1a6b		Fut9	Mylip	D630024D03Rik		
Edar	Ftcd		Car4	Dedd2	F2rl3		
Nfam1	Extl1		Gm7457	Cenpt	Cnnm2		
Usp27x	BC035947		Ugt3a2	Mbd1	Rdh16f2		
Sp110	Dnph1		F13b	Il7	Angpt1		
Ch25h	Grin3a		Slc22a12	Ube2l6	Ndufs1		
Adamts8	Gfra4		Cyp2j11	Eno3	Ddc		
Efhd2	Aspg		Aspn	Arl4d	Syp		
Tes	Gm10787		Slc17a3	Nectin4	Clec11a		
Rab32	Scd1		Tmem27	Pim1	Vstm2a		
Themis2	Aass		Baat	Snhg12	Sun3		
Ccr2	Mroh7		6330410L21Rik	Gm32031	Shank2		
Mab21l3	Spag4		Ak4	Ttc39b	Slc39a8		
Myc	Cfap126		Pdzk1	Gm15728	Gm19461		
Oser1	Car9		Slc10a5	Wars	Asb9		
9430076C15Rik	Vwce		Kl	Klk1b5	Pclaf		
Uspl1	Abhd1		Slc34a1	Nucb2	Morn2		
Stat3	Gm45111		Slc6a20b		Hsf2bp		
Hsf4	Spr-ps1		Gm28438		Shisa3		
Ugcg	Bhmt2		Keg1		Gm14539		
Irf9	Fitm1		Egf		Steap3		
Pip5k1a	Uroc1		Gm15318		Wscd1		
Rgs10	BC089597		Btnl9		Col1a1		
Arl5c	5830473C10Rik		Ugt2b38		Apoo		
Ripk2	Hba-a1		Aadac		Sall2		
Rabgef1	Upb1		Slc39a5		Slc4a4		
Taf1a	Prok1		9030619P08Rik		Kcnj1		
Arhgap9	Gm15348		Slc22a8		Podxl		
Sbsn	Pzp		Cndp1		Ptgr2		
Ammecr1	Gm44810		Pde6a		Has3		
Cd300a	Ces1d		Aplnr		Syt3		
Gtse1	3110045C21Rik		Cpne4		Gm14399		
Tmem43	Tpm3-rs7		Dpys		Dhcr24		
Pea15a	Bco2		Hao2		Scn2b		
Parvg	Haao		Fam151a		Ddn		
Cd72	Adrb3		Lrp2		Itgb8		
Tlr4	Ces1f		Gm37824		Erbb4		
Tnfrsf13b	Gm6614		Gatm		Pcx		
Akr1b8	BC024386		Cyp2j5		Wisp1		
Adm	RP24-282C4.11		Acmsd		Ackr3		
Fosl2	Corin		Pah		Pi15		
Cysltr1	Tbx10		Gabrb3		Qprt		
Rasal3	Olfr1393		Ppp1r1b		Heyl		
Btk	Col6a6		Slc22a22		Ldlr		
Unc13d	Nxph4		Miox		Wnt5b		
Aen	Dnase1		Serpina3b		Pdzrn4		
Cd276	Inmt		Lrrc3b		Klkb1		
Gm13398	Mogat1		Nepn		Cabcoco1		
Adcy7	Nat8f5		Aspdh		Sema3g		
Zswim4	Ido2		Gm4450		Ccdc3		
Adar	Akr1c21		Slc22a28		Slc6a13		
Traf3ip3	Gm44785		Slc34a3		Spink1		
Lgals1	Cyp2j13		Insc		Col4a5		
S100a11			Gm10681		Nes		
Pik3cd			Slc22a30		Aldh3b2		
Itgax			Pvalb		Mapk10		
4933411K16Rik					Mcee		
Proser2					P3h2		
Rgs14					1700006J14Rik		
Wdfy4					Fam81a		
Ier5					Cyp2d26		
Rel					Slc1a1		
Litaf					Coch		
Zbtb34					Wdr86		

Asns					Angptl2		
Plekho2					C1qtnf2		
Plekho1					Dmtn		
Sde2					Asb2		
Sqstm1					Bex4		
Phf11b					Nrap		
Etv3					Ppp2r2b		
Cdk6					Xylt1		
Lrmp					Cacng4		
Tchp					Khdrbs3		
Hpse					Gm33023		
Relt					Tppp		
4930415O20Rik					Rit1		
Neurl3					Lekr1		
Bin2					Tmem52b		
C4b					Spag6		
Foxs1					Sostdc1		
Dnajb13					Hpgd		
Ptger4					Chdh		
Akr1c13					Rdh1		
Tuft1					Hsbp1l1		
F2rl1					Cdkn3		
Gm13807					Slc38a4		
Pou2f2					Mdh1		
Arc					mt-Nd1		
Lbp					mt-Nd4		
1700017B05Rik					Chst3		
Efcab5					Rnf165		
Smcr8					Mdk		
Lag3					Tro		
Wnk3					Cldn19		
Slc1a2					D630014O11Rik		
Sh3bp2					Ptn		
Srgap1					C1ql3		
Nectin2					Colgalt2		
Tbc1d9					Nrgn		
Tbc1d10c					Mgat3		
Eif1a					Kcnn1		
Traf2					Depdc1b		
Gm6548					Plekhb1		
Ltbp2					Trac		
Spn					Kcnn3		
Gm1966					Shc2		
Mtf1					Slc7a7		
Mdm2					Hmgcs1		
Adgre4					Adamts12		
Zfp141					Mki67		
Mmp19					Igfals		
Tnnc1					Nol3		
Fgl2					Gm11814		
Snx16					Pcdh12		
Cpne8					Rps6ka6		
Ptafr					Ogdhl		
Prune2					Acss3		
Mpzl3					Sgip1		
Cd40					Sugct		
Tirap					Cetn4		
Srxn1					Dpp4		
Lacc1					Bdh1		
Plekha4					Rbfox1		
Mvp					Mylk3		
Hcls1					Tmem54		
Gm11131					Mccc1		
Zfp677					Top2a		
Scara3					Stc1		
Fam13b					1810014B01Rik		
Msi1					Lrriq3		
Nup210l					Cd248		
Tlcd2					Myct1		
Psrc1					Tril		
Phf23					Syt13		
Cdkn2b					Slc3a1		
Gpatch3					Rcn3		
Tifab					Wnt5a		
Mob3a					Gimap4		
Micall2					Ppp1r1a		

Hspb8					Slc12a3		
Nfkbiz					Lrrc4c		
Orc1					Unc5c		
Kbtbd8					Wnt11		
Ikbke					Cdk5rap1		
Bak1					Slc16a12		
AI504432					Cldn10		
Etaa1					Clrn3		
AI662270					Fgf10		
Ncf2					Cbr1		
Ogfr					1190007I07Rik		
Zfp992					Prkar2b		
Ctsc					Pcbd1		
Mex3a					Gm13111		
Zfand2a					Lama4		
Suv39h2					Kcnmb1		
Gabrp					Ntf3		
Ubap1					mt-Nd2		
Cstb					Gcm1		
Speg					Lamc3		
Lins1					Ptger3		
Ptgir					Gm29683		
Arhgap28					Spata17		
Bcl9					Crb2		
Dock10					Slc5a12		
Cxcl16					Cryzl2		
Lpxn					Hspa12a		
Fgfr1op					Sfrp4		
Lck					Mmd2		
Rlim					Slc5a9		
Cd300ld					Ndrg4		
Trim21					Igf1		
Smg5					Sult1c2		
Slc20a1					Gm17767		
Wfdc17					Cdh11		
Arhgef5					Tmed6		
Eif2ak2					P2rx1		
Tubb6					Gucy2g		
Tmem140					Col15a1		
Map7d1					Slc14a1		
Cbr3					Aldoc		
Tmsb10					Anks4b		
Cyp1b1					Klhl4		
Prkcb					Itm2a		
Zfp189					Pnmal2		
Map3k13					Col3a1		
Ppan					Chst1		
Elf4					1110019D14Rik		
Ddx52					Gna14		
Kctd10					Unc13c		
Ttc39a					Mettl15		
Src					Kcne1l		
Cttnbp2nl					Ces2b		
Nrip3					Lmx1b		
Baz1a					Rhpn1		
Fermt3					Kcnf1		
Rnf213					Slc9a3		
Atp10a					Smarca1		
Pamr1					Slc14a2		
3110043O21Rik					Slc2a13		
Ccnf					Dio2		
Sbno2					Mgl2		
H2-M3					Akr1b3		
Syt2					Fabp3		
Zbtb39					Tldc2		
Ccl6					Habp2		
Gm44751					Snca		
Hic2					Glod5		
Dpysl3					Acot3		
Rras2					Dzip3		
Mak16					Mrgprf		
Rassf4					Zfp931		
H2-Ab1					Fmo1		
Parp8					Rnf182		
Arf2					Ankle1		
Ifi35					Tal2		

Bspry					Slc12a1		
Actn1					Emid1		
Zfyve26					Gm11992		
Pdlim7					Tmem169		
Rhbdf2					Glt8d2		
Nr4a3					Enpep		
Lrrn4cl					Nphs1		
Mapk6					Nkx3-1		
Myd88					Gm10522		
Ccdc137					Mest		
Rap2b					Vpreb1		
Tuba1a					Spag5		
Ankrd6					Dnah11		
Lyl1					Acnat1		
Ypel2					Fam69b		
Cpeb1					Rgs9		
Cgn					Ckb		
Gdpd5					Pm20d1		
Frmd6					Ugt2b5		
Ramp1					Gm17546		
Gsdmd					Prodh2		
H2-K2					Gm43429		
1810013L24Rik					Myom2		
Dclre1b					Sptssb		
Plag1					Galm		
Blcap					Vipr1		
Mb21d1					Gpc5		
Ddx58					Ephb1		
Sowahb					Itga8		
Mctp2					Tll1		
Gpatch4					Ano5		
Serpinb6a					Gm45847		
Nop58					Elovl2		
Tnfrsf10b					Halr1		
Lonrf3					Mfsd4b3		
H2-DMb2					Mfap2		
Slc35e4					D130043K22Rik		
Fam83g					Col6a3		
Cmtm3					Dlg2		
Il1rl2					Npy		
Gxylt2					4732465J04Rik		
3110001I22Rik					Crispld1		
Cyth4					Tdrd5		
Defb42					Hsd17b14		
Hmha1					Higd1b		
Sox4					Ndufa4l2		
Frk					Vcan		
Fam167a					Gm11789		
Esco1					Slc26a7		
Plekhf2					Gm45495		
Marveld2					Ranbp3l		
Kif1a					Spp2		
Tifa					Gm33543		
2310022A10Rik					Gm43800		
Akr1c12					Gm19541		
AA474408					Xpnpep2		
Lcp1					Ugt3a1		
Klrg2					Gm5524		
Scn7a					Gm17634		
Ubtd2					Slc6a12		
Plekhm3					Gm12002		
P2ry6					Sfrp1		
Pnrc1					Ighm		
Ptprc					R3hdml		
Ifngr2					Tmem100		
Phf11c					Fndc5		
Sesn2					Gm14161		
Pwp2					Ngfr		
Cnksr3					Rph3a		
Plch1					B3galt2		
Tcp11					Abca13		
Epha2					Pnpla3		
Il6ra					Lum		
Hdac9					Slc2a2		
Dpf2					1110032F04Rik		
Il33					Gdf10		

Dcbld1					Spa17		
Nupr1					Slc10a2		
Zfp143					Srpx2		
Rab7b					Gm39078		
Slc31a2					Slc26a1		
Bcl2l1					Slc15a2		
H2-DMa					Stab2		
Tdrkh					Cyp2c23		
Prr15					Phyhip		
Trim25					Adam12		
Matk					Mtfp1		
Cdkn1c					Eddm3b		
Psmb9					Postn		
Capg					Tfpi2		
Mtf2					Hsd3b2		
Pf4					S100b		
Tert					Smpx		
Axin2					Slc10a1		
Mycl					Prlr		
Nr2c2ap					Adamts17		
Sp140					Mfsd4b5		
Fbxo45					Fat3		
Fam109b					Wfdc16		
Zfp410					Camk1g		
Vtcn1					Atp4a		
Plekha2					Gm6878		
Arid3a					Gm37296		
Pdgfb					Slco1a6		
Pitx2					Lypd2		
Pi4k2b					Csrnp3		
Zfp54					3110099E03Rik		
CAAA01180111.2					Kcna6		
Taok3					Cntn1		
Smurf1					Elfn1		
Ptpre					Nrep		
Hn1					Zfp125		
Yy1					Dpt		
Cdh24					Gpr37		
Tchhl1					Mansc4		
Ttll7					Spta1		
Igf2bp3					Gm42397		
Orai2					Gm12326		
Tsr1					Itih1		
Kat7					Igfbp5		
Maml1					Tdg-ps		
Psmb10					Serpina6		
Il4ra					Clec2h		
Mef2d					Slc7a12		
Irf1					Mro		
Dcn					Gfra2		
Sfmbt2					Retnla		
Fgd6					Nphs2		
Nrg1					Gm853		
Slc7a1					Cpn1		
D130040H23Rik					Hes2		
Grwd1					Lrrn2		
Stat1					Tmem207		
Zfp954					Aadat		
Csrnp1					A4gnt		
Lrig1					Gm2115		
Ang					Meox2		
Elf3					2410003L11Rik		
Zfp518a					Gm12892		
Rasa2					Gm16010		
1700024P16Rik					Slc22a7		
AA414768					Gm45650		
Nhlrc3					Tnn		
Tppp3					Slc22a19		
Arpc1b					Chil4		
Ell					Mep1b		
Ifitm1					Cck		
Map4k5					Cox8b		
Zfp655					Ccdc170		
Leo1					Nccrp1		
Utp15					Gm32688		

B230307C23Rik					Kap		
Zcchc10					Sycn		
Ccdc120					Rdh19		
Rnase4					Slc22a29		
Slit3					Slc7a13		
Igf2bp1					Slc22a27		
Cd2ap					Nr2e3		
Jazf1							
Stk17b							
Cirh1a							
Cx3cl1							
Ifitm3							
Marcksl1							
Axl							
Styk1							
Impg2							
Smyd5							
Ptrh1							
Zfp558							
Zfp583							
Gpr183							
Mmp12							
Apaf1							
Nip7							
Casc1							
Nrf1							
Zxdc							
Ccdc112							
Rbm43							
Nck1							
1700016C15Rik							
Rnf121							
Lrp8							
Snx11							
Arid5b							
Ppp1r12b							
Gstp-ps							
Traf1							
Cd74							
Tmem158							
Vim							
Aplp1							
Psme2b							
Eif6							
Tex10							
Cenpj							
Zbtb42							
Atf1							
Nup62							
Rab40c							
Exog							
Slc39a6							
Atg14							
Gxylt1							
Hn1l							
4833422C13Rik							
Unc93b1							
Nkd2							
Gm20559							
Fbxl22							
Mapkapk3							
Wbp11							
Dsel							
Clic1							
Nsrp1							
Morc3							
Gnl3							
Tbrg1							
Gla							
Brd2							
Mex3c							
Tsc22d1							
Vps37c							
Zfp935							
Pramef8							
Rab15							

Fam104a							
Zfp995							
Rps6kc1							
Vopp1							
S1pr2							
Cdc34							
Mettl2							
Casp8							
Pprc1							
Gpr35							
Arhgap15							
Mast4							
Chmp4c							
Ube2g2							
Cry1							
Epc1							
Utp23							
Gm17435							
Tox4							
Haus3							
Nmi							
Samd4b							
Prr5l							
Cdk5r1							
Rbm11							
Zfp667							
Nfkbib							
Dgkg							
Gins3							
Zfp64							
C030015A19Rik							
Ak6							
Traf6							
Mcl1							
Fem1c							
Snx30							
Bid							
AU020206							
Slc25a24							
Rinl							
Pcdhb9							
Lamb3							
AI597479							
Oas1c							
Phldb3							
Mob3c							
Ppp4r2							
Tbpl1							
Btbd19							
Neto2							
Itgb2							
Ccm2							
Krt8							
Gm9892							
Zfp280b							
Bcl10							
Itpkc							
Tfe3							
Grk5							
Ampd3							
Ccdc149							
Tpm1							
Ihh							
Polr3d							
Hoxa1							
Poldip3							
Cyp4v3							
Nepro							
Kif5c							
Zfp605							
Rnf19a							
Zdhhc18							
Top3a							
Mis12							
Zfp105							
Fbxo6							

N4bp1							
Dgcr8							
Med4							
Trim45							
Trim16							
Pknox2							
Gclc							
Fam118a							
Atf4							
